# Training programs in preclinical studies. The example of pulmonary hypertension. Systematic review and meta-analysis

**DOI:** 10.1371/journal.pone.0276875

**Published:** 2022-11-15

**Authors:** Magdalena Jasińska-Stroschein

**Affiliations:** Department of Biopharmacy, Medical University of Lodz, Lodz, Poland; Vanderbilt University Medical Center, UNITED STATES

## Abstract

**Background:**

Exercise and cardiopulmonary exercise testing are essential in the evaluation of physiological, biochemical, and molecular responses in the experimental studies on chronic diseases such as diabetes, heart failure and hypertension. The exercise tolerance and seem to be a valuable contribution to the experiments that are performed in animal models of pulmonary hypertension (PH), as well. The current survey uses detailed quantitative analyses to assess the advantages of exercise training programs performed in preclinical studies based on outcomes such as exercise capacity, cardiopulmonary hemodynamics, and mortality.

**Methods:**

Articles were identified through search engines in the online electronic databases Pubmed/Medline, Web of Science following the PRISMA Protocol. Studies conducted between 1991 and 2022 without language restrictions were included in this study. Heterogeneity was assessed using the Cochrane Q-test and I2 test statistics. Subgroup analysis was employed with evidence of heterogeneity. Quality assessment was carried out using SYRCLE’s risk of bias tool for animal studies. Publication bias across studies was determined using the funnel plot and Egger’s regression test statistic.

**Results:**

The available protocols typically included treadmill running, swimming, and voluntary wheel running with a different series of intensities, times and durations; these were also used in studies examining the efficacy of chronic training programs. In 66 interventions, PH induction reduced exercise endurance by half compared to healthy subjects, while exposure to tested medical agents normalized exercise capacity. The other 58 interventions demonstrated the advantages of various exercise training programs for PH. Induction of PH reduced exercise endurance by half compared to healthy subjects (R = 0.52; 0.48 − 0.55 95%CI; P<0.0001; I2 = 98.9%), while the exposure to tested medical agents normalized exercise capacity (R = 1.75; 1.61 − 1.91 95%CI; P<0.0001; I2 = 97.8%).

**Conclusion:**

Despite a wide spectrum of study protocols to measure exercise endurance in animals with PH, there is a significant correlation between worsening of exercise-related parameters and PH development, manifested by alterations in haemodynamic and remodeling parameters. Familiarization with exercise, training program schedule, method used for PH induction, or detailed training parameters such as slope, exercise intensity or individualization, can influence the final outcome. This in turn can impact on the diversity and reproducibility of results being obtained in particular experimental studies.

## Introduction

Exercise and cardiopulmonary exercise testing are essential in the evaluation of physiological, biochemical, behavioural and molecular responses in the experimental studies on chronic diseases such as diabetes, heart failure, hypertension and pulmonary hypertension (PH). PH is characterized by an increase of pulmonary vascular resistance and pulmonary arterial pressure, leading to right heart insufficiency. Although therapy for PH has evolved progressively in the past decades, patients demonstrate unsatisfactory survival and typically remain symptomatic, with impaired quality of life and exercise capacity [1]. Therefore, a key role in the evaluation of PH patients, together with symptom monitoring, assessment of functional class, hemodynamic parameters and biological markers, is played by exercise tests [2]. Such tests include the repeatable six-minute walk test (6MWT), standard treadmill exercise test performed using low-intensity exercise protocol, cardiopulmonary exercise test (CPET) performed by gas-exchange measurement, exercise test performed simultaneously with Doppler echocardiography, or right heart catheterization [1]. The greater understanding of the epidemiology, pathogenesis, and pathophysiology of PH has led to an explosion in research in the field of pulmonary vascular disease over the past few decades; the previous reports revealed hundreds of treatments to be effective in a variety of animal models. Exercise intolerance, being the characteristic symptom of PH, appears to be a valuable contribution to most experiments performed in animal models of PH. Numerous animal exercise research protocols have been established to study the impact of PH on animal cardiovascular function. Similar protocols are intended for testing novel therapies for PH, including exercise training programs, as well. In most cases, they involve treadmill running, swimming, and voluntary wheel running with a series of intensities, times and durations. Pooled quantitative evidence comparing the effect of such interventions is scarce.

The aim of the current survey is to quantitatively summarize the effects of different types of regular aerobic and anaerobic exercise interventions on pulmonary hypertension. An emphasis was placed on the settings of training programs adopted to PH animals, their beneficial effects on exercise tolerance and disease development as well as major determinants of study outcomes. Detailed information being addressed with PICO is provided in next section. The questions addressed by the study were as follows: (a) Is there any relationship between the results of animal exercise endurance and the severity of the pulmonary hypertension? (b) Do the particular training programs improve exercise intolerance and normalize pulmonary hemodynamics and hypertrophic changes in animal models of PH? (c) If so, are there any additional factors that can determine the outcomes–exercise endurance and PH-related lesions? (d) If these factors exist, how can such influence be quantified? Finally, the practical implications of the present findings are to raise awareness of exercise programs for treating PH, and the need to improve the method standardization in relation to clinical settings.

## Material and methods

### Inclusion and exclusion criteria

The search included experiments that were performed on different models of pulmonary hypertension (PH) *(population)*, where animals could have been exposed to several exercise training programs to prevent or reverse the disease *(intervention)*. The animals underwent a wide spectrum of haemodynamic, echocardiographic and histopathologic assessments, and various exercise tests *(outcome)*.

All experiments included a cohort of sedentary (PH sed) or training (PH train) animals with PH. A cohort of healthy sedentary animals (Sham) or sedentary animals with PH were used as a *comparator*. The exclusion criteria were as follows: experiments on pregnant, newborn animals or foetuses, studies in which acute PH was induced or several medications were examined after their acute administration, and studies that lacked data, such as the number of animals.

### Data extraction

PubMed, and Medline-Ebsco databases were searched using the keywords (MeSH) (‘pulmonary hypertension’) AND (‘rat’ OR ‘mouse’ OR ‘mice’) AND (‘exercise’ OR ‘capacity’ OR ‘exhaustion’ OR ‘fatigue’ OR ‘treadmill’ OR ‘swimming’ OR ‘running’ OR ‘training’) from January 1991 to March 2022. The following data were recorded: animal species, including genetic modifications if needed, the animal model of PH (inductor, dose, induction period), name of tested substance to prevent or reverse PH (agent, dosage, time and route of administration), details from exercise protocol, main outcomes–alterations in PH-related parameters that were measured invasively: right ventricle mean/systolic pressure–RVP/RVSP, in mean pulmonary artery pressure–mPAP, or non-invasively: PAT, PAAT, TAPSE, AT/ET or CO, pulmonary artery (PA) remodelling, expressed as medial wall thickness (%), or degree of muscularization, and/or right ventricle hypertrophy (Fulton index–usually expressed as RV/LV+S ratio), as well as alterations in animal exercise capacity (time to exhaustion, distance, maximal oxygen uptake–VO2max, time to maximal oxygen uptake–VO2max), data about initial and final animal body weight, and animal mortality across the study. The outcome measure included mean, SD or SEM, and number of animals per group. If the number of animals was reported as a range (e.g., 6–11), the lowest number was used, if the authors did not provide the number of animals that finished study, the initial number of subjects (at randomization) was used. Two independent reviewers (MJ-S, PP) performed the literature search, data extraction, and methodological grading. Disagreements were resolved by consensus.

### Quantitative data synthesis

The analyses were conducted using STATISTICA 13.1 software. For each pairwise comparison between two treatments, the relative effect was calculated with a 95% confidence interval (CI). When the results of more than one comparison, i.e. tested agent *vs*. Vehicle, were reported in one study, e.g. due to different experimental conditions (e.g. animal model or method used to assess exercise capacity), such a comparison was regarded as a separate ‘intervention’. In cases where the outcome measures were reported as median and range or 95% CI, mean and SD values were estimated according to Wan et al. (2014) [3]. The effect size for the sedentary animals with PH (*PH sed*) in relation to healthy subjects was assessed according to net changes in measurements,

$$D=\bar{X_{PH\;sed}}-\bar{X_{healthy}},\;or$$ (1)

was expressed as response ratio,

$$R=\frac{\bar{X_{PH\;sed}}}{\bar{X_{healthy}}},$$ (2)

For protocols that included exercise training programs, the effect size for the training animals with PH (*PH train*) in relation to their sedentary counterparts (*PH sed*) was assessed according to net changes in measurements,

$$D=\bar{X_{PH\;train}}-\bar{X_{PH\;sed}},\;or$$ (3)

was expressed as response ratio,

$$R=\frac{\bar{X_{PH\;train}}}{\bar{X_{PH\;sed}}},$$ (4)

where D–difference in means; R–response ratio; $\bar{X}$–mean response in the particular group of animals

Animal weight gain or loss was analysed based on Eq (1). Alterations in exercise capacity or PH-related parameters among healthy and sedentary animals with PH were calculated using Formula (2). Alterations in exercise capacity between sedentary animals and these that received exercise training programs for PH were calculated using Formula (4). The increased response ratio (R) values indicate worse PH-related parameters (↑ RVSP, RV/LV+S, or artery remodeling), and better exercise endurance (↑ time to exhaustion, distance, VO_2_max, etc.). The Formulas from (1) to (4) were used for comparative analyses of publication bias.

Subgroup analyses were pre-defined in the protocol with the purpose to assess the influence of selected variables on the outcome, for example, the method used to test exercise capacity, familiarization with exercise equipment, exercise training program or the method to introduce PH. A random-effects model was used to compensate for the heterogeneity of studies. Heterogeneity was quantitatively assessed using Cochran’s Q and I^2^ statistics. A two-tailed P-value less than 0.05 was considered statistically significant.

### Survival analyses

The Kaplan-Meier method was used for survival analyses. The difference in survival between animal groups or established protocols was evaluated using the log-rank test for two groups, or the non-parametric Chi^2^ test for more than two groups.

### Risk of bias and publication bias

Quality assessment was carried out using SYRCLE’s risk of bias tool for animal studies [4]. A ‘Yes’ indicates a low risk of bias, while a ‘No’ indicated a high risk of bias in a specific domain. The risk of bias for papers that did not provide enough information in this area, was defined as ‘Unclear’. This was assessed independently by MJ-S and PP, any differences were resolved by consensus.

Potential publication bias across the studies was examined using a visual inspection of Begg’s funnel plot asymmetry, Begg’s rank correlation, and Egger’s weighted regression. Duval and Tweedie ‘trim and fill’ was used to adjust the analysis for the effects of publication bias.

#### Ethical consideration

This article does not contain any studies with human participants or animals performed by an author.

## Results

### General design of preclinical studies

**Fig 1:**
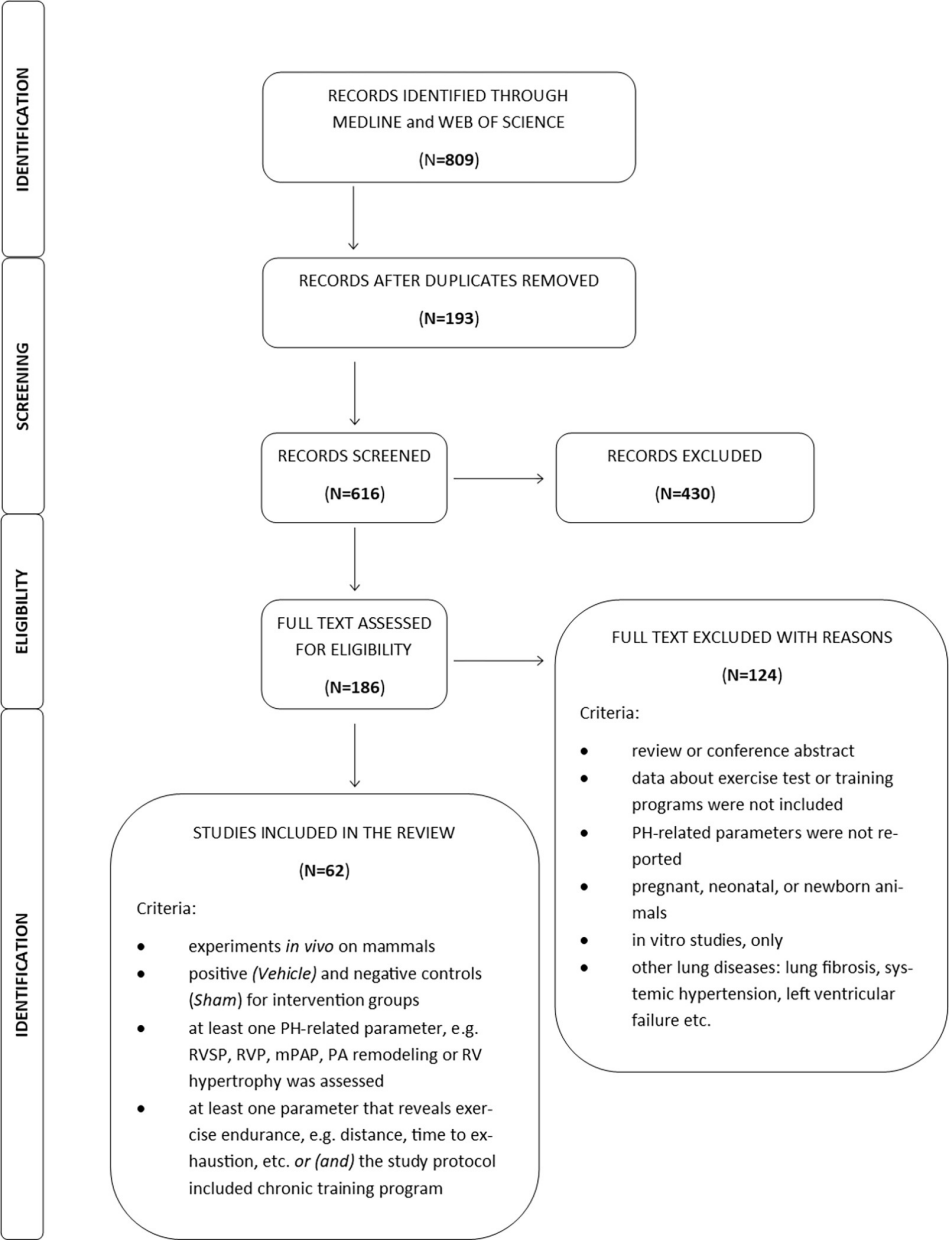
PRISMA flow diagram of the search study.

The search yielded 809 papers. After adjustment for duplicates, 616 studies remained. Of these, after screening the titles and abstracts, 554 articles were found to be irrelevant to the review question and were discarded. The final corpus comprised 62 papers, as demonstrated in the PRISMA flow diagram (Fig 1). The list of papers included into systematic review can be found in Supplementary material.

**Fig 2:**
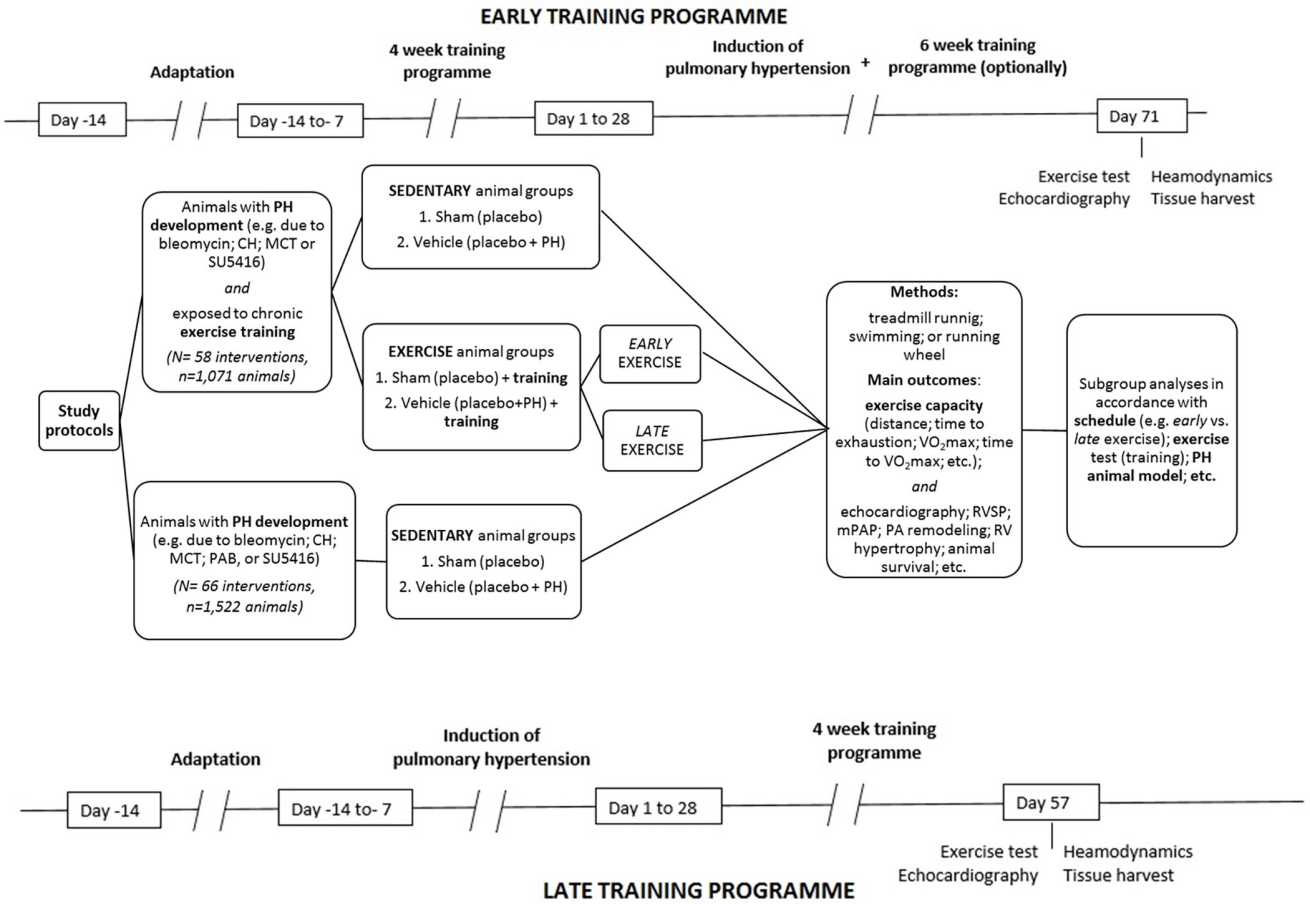
The study flow according to the purpose (exercise training, and/or assessment of animal capacity), some methodological items that characterized the analyzed experimental protocols on pulmonary hypertension, and an example scheme for study protocols for early and late training. CH–chronic hypoxia; MCT–monocrotaline; PA–pulmonary artery; PH–pulmonary hypertension; PAB–pulmonary artery banding; RV–right ventricle; RVSP–right ventricle systolic pressure; SU5415 –Sugen 5416; VO_2_max–maximal oxygen uptake.

The term *intervention* refers to a separate comparison between healthy and sedentary animals with PH, or between sedentary and training animals–both with PH; they could differ in PH model, exercise test, training program, tested agent or dosage schedule. Thus, from 62 studies, 124 interventions were identified. Fig 2 demonstrates the study flow, according to the type of animal model of PH, the exercise test protocols, or the training program identified in the experimental studies, and included in the analysis.

In brief, PH-related lesions were assessed in 2,593 animals. Fifty-eight interventions concerned the advantages of various exercise training programs for treating PH. Their efficacy was assessed based on observed differences between sedentary subjects and training counterparts with regard to selected haemodynamic and (or) hypertrophic parameters, exercise endurance and mortality. The effects of two main training schedules, *viz*. early *vs* late training programs, were also evaluated. For the purposes of *early* training schedules animals were exposed to chronic exercise training before PH induction; *late* training program meant that the training was started only 14–21 days after PH induction. In other studies, sedentary animals (66 interventions) were exposed to various methods to introduce and treat PH, and their exercise endurance was assessed. In case of the two mentioned schedules, the sedentary and training animals were exposed to a variety of procedures in order to develop PH, such as chronic hypoxia (CH), monocrotaline (MCT) or SU5416 injections, and pulmonary artery banding (PAB) procedure. More detailed information about studies included in the analysis is provided in S1 Table, while the details of exercise tests and training programs used for PH animals are given in S2 Table.

### Exercise tests

**Fig 3:**
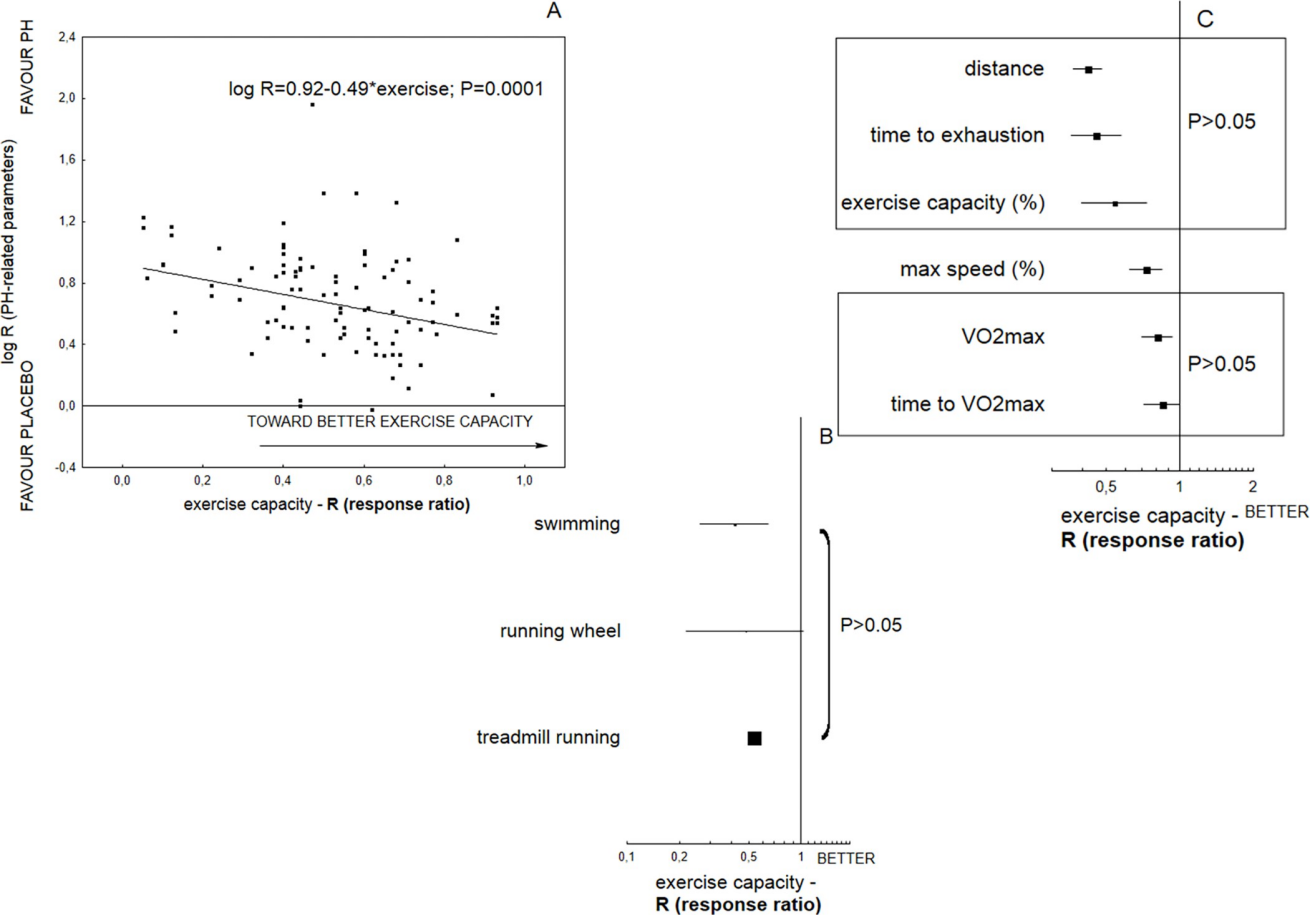
The performance of animals with pulmonary hypertension in relation to methods used for the assessment of animal exercise capacity. The overall effect was expressed as response ratio (R), according to alterations in both hemodynamic (RVSP, mPAP) and remodeling parameters (Fulton index, PA muscularization), as well as animal exercise capacity. The increased values of response ratio (R) reveal better exercise endurance (*n = 1955* animals). A statistically significant Q measure (P<0.05) indicates heterogeneity among two or more analyzed subgroups. Meta-regression plot (*124 comparisons*) demonstrates that worsening of PH-related parameters was significantly correlated with poorer animal exercise capacity (P = 0.0001) (**A**); the method chosen for the exercise tests had no influence on the resultant changes in exercise capacity (Q = 0.59; df = 2; P>0.05) (**B**); among a wide range of parameters that could be detected in the analyzed experiments, a similar exercise capacity (**C**) was observed where the following sets of parameters were considered: distance, time to exhaustion and percentage exercise capacity (Q = 2.1; df = 2; P>0.05) or values of VO_2_max and time to VO_2_max (Q = 0.19; df = 1; P>0.05).

A variety of protocols were established (S2 Table) for the evaluation of exercise endurance; of these, a motor-driven treadmill predominated (85.2% of interventions), with some using a swimming pool (9.8%) or running wheel (5%). In general, the exercise tests were performed once prior to induction of PH, and again at the end of the experiment. In general, induction of PH reduced exercise endurance by half compared to healthy subjects (R = 0.52; 0.48 − 0.55 95%CI; P<0.0001; I2 = 98.9%), while the exposure to tested medical agents normalized exercise capacity (R = 1.75; 1.61 − 1.91 95%CI; P<0.0001; I2 = 97.8%). The poorer exercise capacity observed in the sedentary animals with PH was correlated significantly with worsening of hemodynamic and remodeling parameters related to the disease (P = 0.0001) (Fig 3A and S3 Table). The final result of the exercise test was mainly expressed by the authors as treadmill distance (m), treadmill distance multiplied by body weight (mkg), exercise duration (minutes or seconds), maximal oxygen uptake parameter (VO_2_max) (ml/kgh or ml/ kg^0.75 min), time to VO_2_max (min) or percentage of basal exercise capacity. The measurements were performed until animal fatigue or exhaustion, which was confirmed by loss of righting reflex, cessation of running or when the animals accepted three consecutive electric stimulus as opposed to running (S2 Table). The method chosen for the exercise tests had no influence on the resultant changes in exercise capacity (P>0.05). Among the wide range of tested parameters, distance, time to exhaustion and percentage exercise capacity (P>0.05) or values of VO_2_ max and time to VO_2_ max (P>0.05) yielded similar exercise capacities when measured (Fig 3B and 3C and S3 Table).

According to the regression and subgroup analyses, the animals that were reported to be previously familiarized with the method of measure achieved better results in exercise tests (P<0.0001). Regression analysis indicated that such animals manifested better exercise capacity across the study (P = 0.0003) as compared to other animals (any alterations were not denoted, P>0.05). Animals adopted to the procedure of exercise testing also demonstrated less worsening of PH, especially less pulmonary artery remodeling (P = 0.003). The only deterioration of PH throughout the experiment, expressed by increasing values of hemodynamic and remodeling parameters, was observed in unfamiliarized animals (P = 0.03) (S1 Fig and S3 Table).

### Exercise training programs for PH

**Fig 4:**
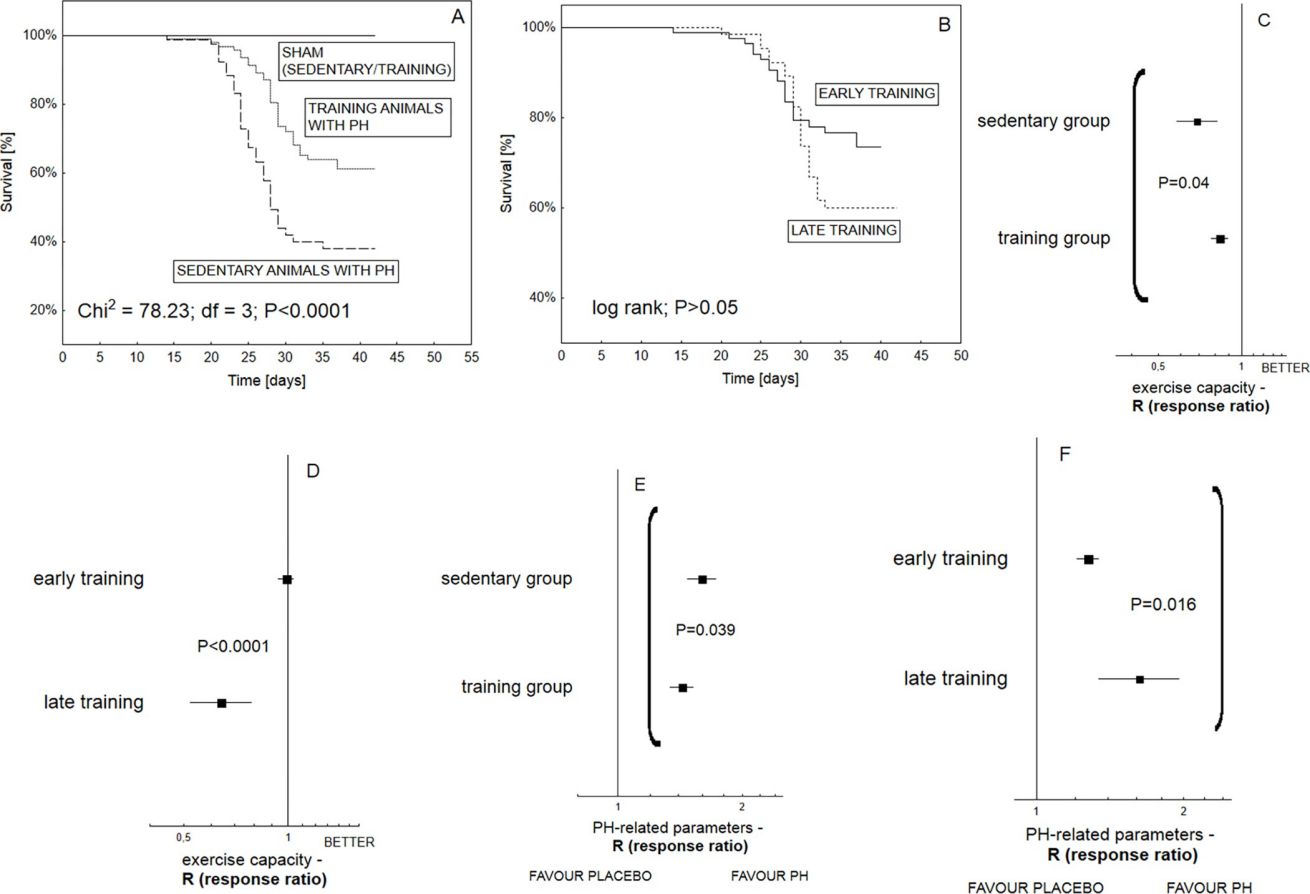
The influence of different exercise training programs on the condition of animals with PH (*n = 1470 animals*). Kaplan-Meier curves demonstrate the significant differences in overall survival among particular study groups (P<0.0001) that were exposed to different exercise training-related schedules (*n = 266 animals*); the mortality of animals with pulmonary hypertension from sedentary groups was higher than those subjected to exercise training (P = 0.0002) (**A**); early training protocol did not improve survival significantly (P>0.05) as compared to these subjects with PH that were exposed to the late training program (**B**), animals developed PH due to injection of monocrotaline (**A** − **B**); tree-plots show that training animals were characterized with significantly better exercise endurance (Q = 4.2; df = 1; P = 0.04) and weaker PH development (Q = 4.3; df = 1; P = 0.039) as compared to their sedentary counterparts (**C**, **E**); early training programs yielded benefits in relation to exercise endurance (Q = 16.7; df = 1; P<0.0001) and PH worsening (Q = 5.85; df = 1; P = 0.016) (**D**, **F**). The overall effect was expressed as response ratio (R), according to alterations in both heamodynamic (RVSP, mPAP) and remodeling parameters (Fulton index, PA muscularization), as well as animal exercise capacity. Increased values of response ratio (R) reveal worsening of PH-related parameters and better exercise endurance (**C**–**F**). A statistically significant Q measure (P<0.05) indicates heterogeneity among two or more analyzed subgroups.

According to data available from experimental protocols, overall survival was found to significantly increase in animals with PH that were exposed to chronic training programs as compared to their sedentary counterparts (P<0.0001) (Fig 4A). Such benefits were accompanied by the improvement of exercise capacity (P = 0.04) and less significant progress of PH, especially in relation to pulmonary artery remodeling (P = 0.039) (Fig 4C and 4E and S3 Table). Although no significant differences in overall survival were observed between animal subgroups that were included into the early vs late training programs (P>0.05), the former demonstrated better results in the exercise test (P<0.0001) and less pronounced RV hypertrophy (P = 0.016) (Fig 4B, 4D and 4F and S3 Table). Similarly, animals that were exposed to chronic exercise training before PH induction (i.e. the *early* training program) manifested less worsening of several PH-linked lesions assessed by echocardiography than those in the late training program (P = 0.006) (S4 Table). The experiments that included early training programs were shorter: a median length of 28 days compared to 56 days.

S2 Fig demonstrates the significant impact of training program on the improvement of PH-related parameters, assessed invasively, according to the method of PH induction. The benefits of exercise training were more apparent for subjects that developed PH due to chronic hypoxia (P<0.0001) than those treated with MCT (P = 0.036).

**Fig 5:**
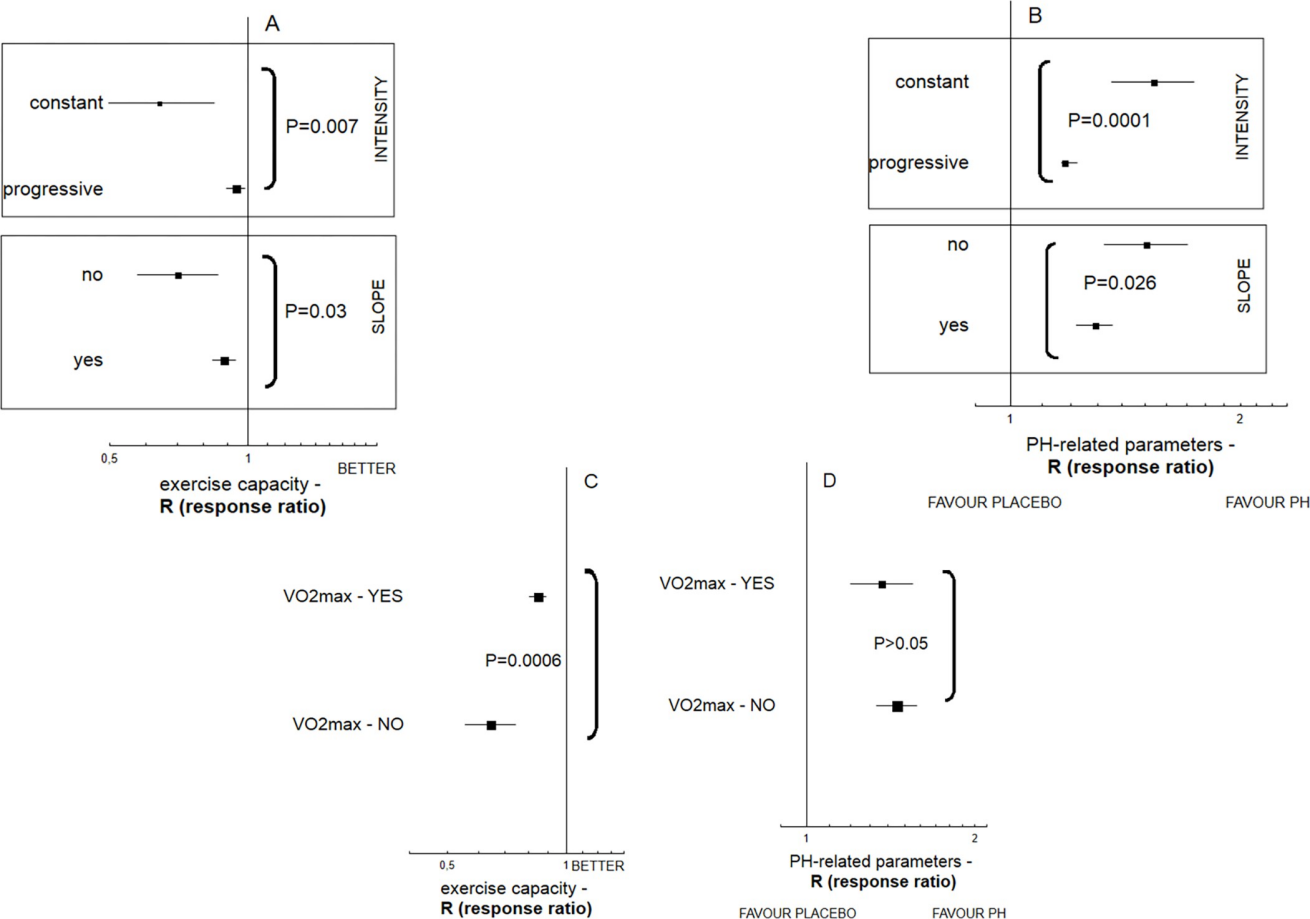
The influence of some methodological items that characterize training programs on the resulting exercise endurance of animals with PH and disease development (n = 753 animals). The overall effect was expressed as response ratio (R), according to alterations in both hemodynamic (RVSP, mPAP) and remodeling parameters (Fulton index, PA muscularization), as well as animal exercise capacity. The increased response ratio (R) values reveal worsening of PH-related parameters and better exercise endurance. A statistically significant Q measure (P<0.05) indicates heterogeneity among two or more analyzed subgroups. Tree-plot for the effect size demonstrates better results in exercise tests that were achieved by animals that were exposed to a chronic training program (treadmill subgroup) that included progressively increased speed (Q = 7.18; df = 1; P = 0.007) and incline (Q = 4.74; df = 1; P = 0.03) (**A**); and when the training intensity was adjusted to VO_2_max parameter, assessed individually (Q = 11.8; df = 1; P = 0.0006) (**C**); increasing intensity and slope were also related to less development of PH-related parameters in training subjects (Q = 16.1; df = 1; P<0.0001 and Q = 4.9; df = 1; P = 0.026, adequately) (**B)**; any significant impact of individually matched training program (adjustment to VO_2_max) was not denoted for PH development (**D**).

Better results in exercise tests were achieved by animals that were exposed to long-term training programs that included incline and progressively-increased speed (P<0.05), and the training intensity was adjusted to VO_2_max parameter, assessed previously (P = 0.0006). Slope and increasing intensity were also related to weaker development of PH-related parameters associated with pulmonary function and vasculature, in training subjects (P<0.05) (Fig 5). S5 Table demonstrates the changes in body weight of animals with PH exposed to exercise training programs. Among animals with PH induced by the injection of monocrotaline, sedentary animals manifested more pronounced body weight loss as compared to their training counterparts (P = 0.005).

### Risk of bias and publication bias

**Fig 6:**
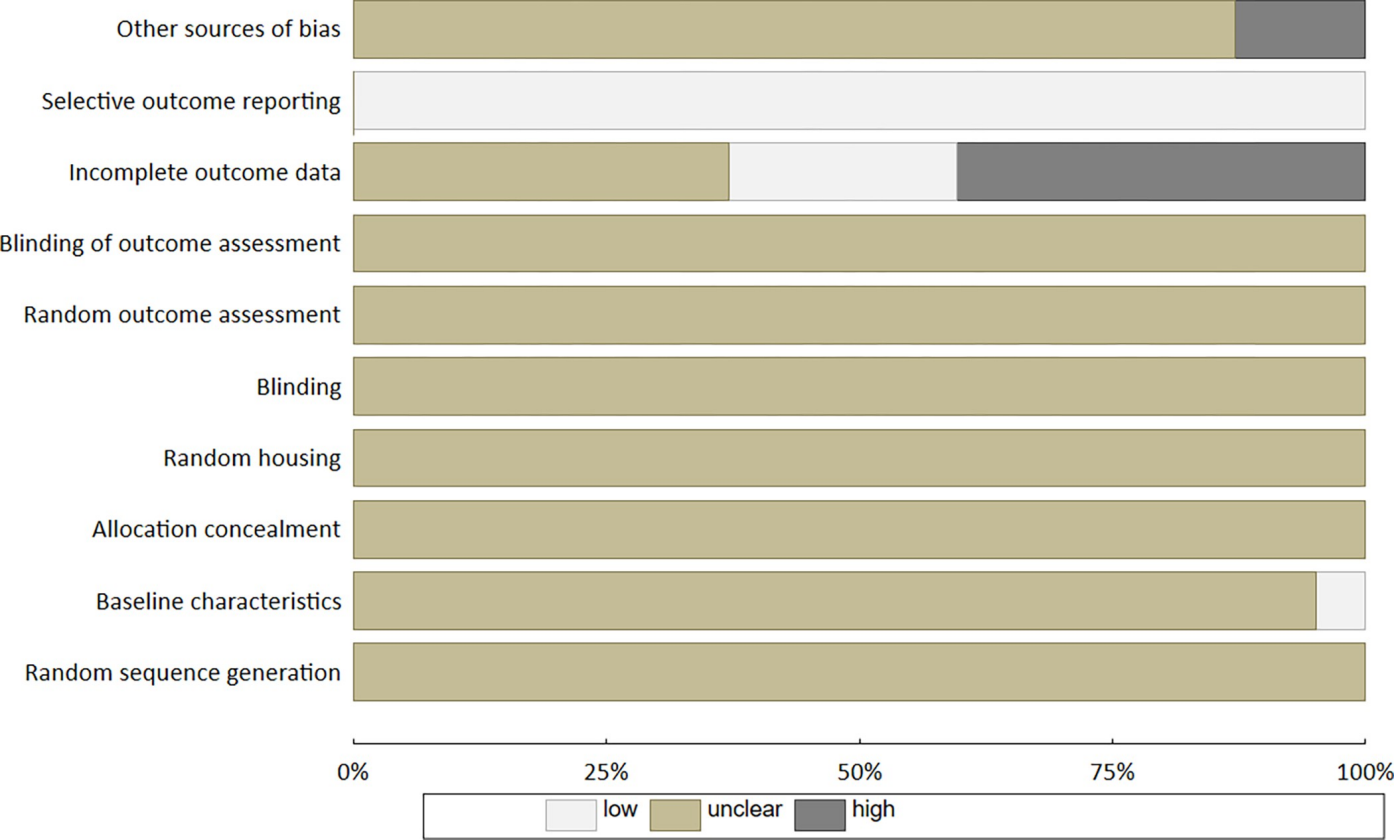
Summary of risk of bias adopted from Hooijmans et al (2014) [4].

The results of the risk of bias evaluation are presented in Fig 6. Forty percent of the studies reported that the animals were randomly submitted to the respective experimental protocols. However, it was not noted which methods were used to generate the allocation sequence, or to house the animals randomly within the animal room (unclear risk of bias). Only one study stated that animals were selected at random for an outcome assessment; however, this did not give sufficient detail to allow such assessment to produce comparable groups (unclear risk of bias). The remaining studies did not provide any information about the randomization process (unclear risk of bias). Only 23 from 62 papers (37.0%) mentioned blinding at any level. They reported that the outcome assessment was blinded, mainly histomorphometric and statistical analyses, however it was not stated how trial caregivers and researchers were blinded from knowing which intervention each animal received (unclear risk of bias). The remaining studies did not provide any information about the blinding (unclear risk of bias). Most papers (95.2%) did not provide the baseline characteristics of the animals (unclear risk of bias). In 22.6% papers, the strict number of animals per group at the beginning and end of the experiment was provided, and such data allowed to assess whether it was completed (low risk of bias); however, in another 25 papers, no information was given regarding the number of animals before the experiment or the number of subjects that finished the study (high risk of bias). In eight trials, other sources of bias were identified: these papers did not replicate the same treatment procedure in the control and experimental groups, new animals were probably added to replace drop-outs from the original population, or the authors did not provide the raw data for results of exercise testing (high risk of bias).

Further analyses were performed to assess publication bias. They involved several subgroups of animals, e.g. sedentary or training subjects in relation to PH-related parameters and exercise endurance (S3 Fig). A fairly symmetrical plot with non-significant Egger test result indicates the absence of publication bias across the studies. Visual inspection and the results of Egger’s test (P<0.05) suggest missing studies and publication bias for the majority of studies, however.

## Discussion

The present systematic review is the first to perform a comparative quantitative analysis of exercise-related tests and training programs used in preclinical experiments on PH. The literature review revealed a wide spectrum of experimental protocols. The heterogeneity levels were generally high; animal studies are often explorative and heterogeneous regarding experimental protocols when compared to clinical trials. Subgroup analyses were used to reduce inter-study heterogeneity: to account for different experimental conditions and examine the influence individual components of the model. In most studies, the effect size indicating changes in PH-related lesions or differences in exercise endurance between animals with PH and healthy subjects was expressed as risk ratio, rather than as net change in the mean values of a particular parameter. This allowed the analyses to be run independently of the units of measurement (e.g., distance in meters, or meters per animal b.w.), the parameters used to express animal capacity (e.g., distance, time to exhaustion, VO_2_max, etc.) or the parameters used to express progress of PH (RVSP, mPAP, PA remodeling, RV hypertrophy). Due to the wide spectrum of calculations and outcomes used in the studies, the results of the detailed analyses concerning particular PH-related lesions assessed during echocardiographic, haemodynamic or histopathological measurements are provided in the Supplementary material. The described protocols used various models of PH induction, such as injections of monocrotaline or SU5416, exposure to chronic hypoxia, genetic modifications, these based on surgical interventions, such as pulmonary artery banding, as well as combinations of such methods, e.g. SU5416 plus chronic hypoxia.

The aerobic exercise training was conducted in experimental studies on rodents with PH. These interventions comprised of running on a treadmill, with one modified with eccentric treadmill training, and a running wheel offered for voluntary exercising. High intensity interval training (HIIT) was performed by rats in one study. The most popular exercise test was based on treadmill running. It was performed in a continuous manner with a fixed or progressively increasing parameters such as inclination, speed, and duration that were different in particular protocols. Exhaustion could be established when the rat was unable to keep pace with the treadmill, even after repeated application of aversive electric stimuli. In such methods, the researcher was required to minimize toenail and foot problems. In some protocols, not-relevant exercise intensity was introduced, and the exercise protocol was designed to ensure that the animals with PH could perform the physical activity even after the development of symptoms of cor pulmonale [5]. Some authors stated that loss of control over locomotion on the treadmill and the reinforcement stimulus can trigger stress responses among experimental animals, and they chose voluntary running for their protocols. In such cases, animals had free access to a wheel and running distance was recorded daily. Such a method also raised the possibility of wheel loading to enhance the effects of exercise. It also allowed the animals to continue exercising when heart failure signs developed, thus better modelling the use of exercise as a treatment in patients with established disease [6].

Another exercise method was the modified forced swimming test. The animals were placed in a cylinder beaker, of defined height and diameter, filled with water. Swimming tests required more rudimentary equipment and allowed to avoid foot injuries however, differential parameters of container, water depth and temperature could also influence the overall result [7].

The presence and extent of a training effect has been assessed primarily using one or more of the following criteria, e.g. endurance capacity and increases in VO_2_max. In a few studies, training-induced reductions in heart rate were measured at rest and during submaximal exercise. Other protocols examined reductions in the blood lactate response during submaximal exercise, or increases in skeletal muscle oxidative enzyme activity. In 45% of the analysed interventions, the maximum walking distance was employed as a measure of the physical fitness. This parameter is regarded to a certain degree as comparable with the 6-min walking distance used in clinical studies to estimate functional capacity in patients with PH [8]. In addition, VO_2_max represents the maximum capacity of an organism to transport and use oxygen and its value reflects the physical fitness of the individual subject; it has emerged as a suitable predictor of mortality in many chronic cardiopulmonary diseases [9]. Current findings indicate that similar exercise capacities were obtained when the following two sets of parameters were measured: the first being distance, time to exhaustion and percentage exercise capacity, and the second being VO_2_max values and time to VO_2_max.

In all experimental protocols, PH induction significantly reduced exercise capacity in sedentary animals as compared to healthy subjects. No significant discrepancies were found between the use of a particular method (e.g. treadmill, swimming or voluntary running wheel) to assess exercise endurance in sedentary animals. This was probably due to the significant dominance of the treadmill (85% of interventions), and the considerable variability in the data for the remaining protocols.

Interestingly, the procedure of animal familiarization with the specific environment and exercise equipment seemed to determine both physical endurance and progression of pulmonary hypertension. Undoubtedly, such habituation can be helpful especially for sedentary animals which usually underwent single physical testing on a treadmill. It helps prevent incidences of foot injuries and decreases the percentage of subjects that might refuse to run and must be eliminated from the experiment. In general, animals should be allowed to run for five to ten minutes per session at low speeds, and the frequency and duration of these sessions must be gauged carefully to avoid training effects [7]. Procedure of adaptation was reported in half of the analyzed protocols; in some studies familiarization process was addressed to all groups of animals, while in others–to the subjects from training groups, only. The process of habitation could take from two to 21 days, with different frequencies in a week (e.g., 3 days a week, 5 days a week, every day, etc.) and durations throughout the day (i.e., from one minute to 1 hour). The speed and incline could be constant or progressively increased, up to 25 m/min. Some authors did not report about details of adaptation. Therefore, the conclusions about positive effects of familiarization with exercise equipment seemed to be biased by a heterogeneity of particular experimental protocols. The assumption that adaptation process in these studies produced similar effects to these in chronic training programs, and improved symptoms associated with exercise intolerance in PH requires further investigation. Nevertheless such phenomena emphasizes that authors should design their protocols with caution, also in relation to the procedures of adaptations.

Chronic exercise training programs were found to improve overall animal survival, as well as exercise endurance, and exerted some cardioprotective effect based on pulmonary artery remodeling in animals with PH. It can be speculated that an improvement of the pulmonary vasculature dysfunction induced by exercise training can further diminish right ventricle afterload. Previous studies of chronic exercise effects found it to have mixed impact on hemodynamics. In the current survey, no significant improvement was observed for the most commonly-reported hemodynamic parameters, such as right ventricle systolic pressure (RVSP) or pulmonary arterial pressure (PAP). In three quarters of exercise training programs, a monocrotaline (MCT) model was used to induce PAH. Although this results in a higher mortality rate due to the toxicity of MCT, several PH-related lesions are induced, such as cardiac remodelling, functional deterioration, pulmonary vasculitis, narrowing or obliteration of pulmonary vessels, and progression to right-sided HF [10]. In some protocols animals were exposed chronically to hypoxia (10% oxygen). This experimental model has emerged as highly reproducible within one animal strain, but it only induces a mild form of the disease, such as relatively small changes in small vessel muscularization; this could explain the weaker results of exercise training introduced for animals exposed to MCT, when compared with chronic hypoxia. Exercise training programs also prevented excessive body weight loss in MCT-treated animals; such loss is typical in this group due to severe pulmonary vascular disease.

Besides the method of PH induction, another item that could differentiate the obtained results was the training schedule. The early exercise protocols were able to attenuate more the development of RVH and improve exercise capacity, than the late training programs, i.e. where the training was started only 14–21 days after PH induction. Also analysis of echocardiographic data, including TAPSE as a classical index of RV systolic function, found that animals starting exercise training before PH induction demonstrate significant amelioration of their cardiac function and morphology. This could confirm that greater improvements are obtained when exercise is initiated in the early stages of the disease. In a few early training protocols, the exercise was interrupted after the induction of the disease. It can be suspected that for better clinical status, exercise should be continued throughout the disease course. However, data related to the outcomes in such protocols were scarce, which made it impossible to perform further detailed comparisons.

Regardless the training schedule (early *vs* late), in general, animals were exercised on the treadmill from 30 to 60 minutes, five days a week, with a running speed of 10–30 meters per minute. Incline and progressively increased speed (2–5 meters/minute) resulted in less PH development and promoted better results in exercise tests performed at the end of the experiment. In addition, inclinations between 15 and 35° were discovered to result in the highest level of maximal oxygen consumption. Individualized exercise protocols, where the training intensity was adjusted to VO_2_max, demonstrated less worsening of the disease. In such cases a moderate exercise intensity corresponding to ≈ 60% of VO_2_max was used, and this intensity can be compared with the recommendations for patients with severe disease [11]. For this reason, a setup allowing personalized control and quantification of the study outcome, e.g. a treadmill equipped with a spiroergometer, can be valuable option for such experiments.

In one study, the advantages of traditional continuous exercise training program (CExT) were compared with high intensity interval training program (HIIT). The former protocol was characterized with uninterrupted steady-state running that was performed with treadmill speed and incline set to elicit an intensity of 50% of VO_2_ reserve. The session duration was progressed from 30 to 60 minutes. For subjects assigned to HIIT, sessions began with a 6-min warm up at 50% VO_2_ reserve and then proceeded into five five-minute cycles of alternating high- and low-intensity intervals with a total duration of 30 minutes. The benefits were more pronounced for a HIIT program that promoted a decrease in pulmonary pressure and attenuated RV hypertrophy and dysfunction [12]. Due to the different exercise modes, the results from HIIT were not included into further analyses.

In general, beneficial effects induced by exercise training might involve preventing skeletal muscle wasting and dysfunction, pulmonary vascular reactivity or pulmonary gas exchange and preventing hypoxemia during the exercise test. Mechanisms underlying such exercise-induced protection can be related to the prevention of mitochondrial damage, fibrosis, decreased oxidative capacity, inflammation or neurohumoral activation. A more detailed summary of the expected advantages of exercise training, which were observed in a number of experimental models of PH, and their mechanistic background, is provided in Table 1 [13–21]. Table 2 summarizes the major findings from existing preclinical studies in relation to clinical ones [1, 22] with an emphasis on the settings of training programs adopted to PH subjects, their beneficial effects on exercise tolerance and disease development as well as major determinants of study outcomes.

**Table 1:** Summary of potential mechanisms underlying the beneficial effects of chronic exercise training in animal models of pulmonary hypertension.

Target	Mechanistic background	Potential advantage of EXT	Reference
Assembly of mitochondrial SC	Formation of mitochondrial ETC supercomplex consisting of complex I and varying copies of complexes III and IV has emerged as an important regulator of mitochondrial respiration.	Increased formation of mitochondrial ETC supercomplex promotes more efficient respiration, improving mitochondrial function and VO_2_max.	[13]
Fiber type distribution	Beta/alpha-MHC ratio reflects alteration in amounts of myocytes, extracellular matrix and collagen, and the myocyte contraction rate. Accumulation of fibrosis has a negative impact on cardiac function, cardiac stiffness, arrhythmias and impairs the diffusion of oxygen to cardiomyocytes.	Reduced beta/alpha- MHC ratio and fibrosis can contribute to the improved cardiac function. The anti-fibrotic effect of exercise may be related to its anti-inflammatory proprieties, and to the down-regulation of ET-1 mRNA of in RV.	[14–16]
Calcium transport proteins	Reduction in SR Ca2+ uptake results in systolic dysfunction as well as increased cytosolic Ca2+ level and susceptibility to apoptosis. In heart failure, SERCA2a activity and its expression are decreased.	Increased expression of SERCA2a in the RV promotes better RV contractile function.	[17]
Inflammation	Several pro-inflammatory cytokines are related to inflammation, oxidative stress, and apoptosis in the RV.	Decrease in circulating and tissue levels of TNF-α, IL-10, NF-kβ, MMPs and caspase-3, as well as the expression of TWEAK.	[16, 18]
ROS and antioxidant capacity	Oxidative stress plays a central role in the development of PAH and RV remodeling. It promotes increased lipid damage in skeletal muscle cells, accumulating lipid peroxides and destabilizing effects on cell membranes.	Reduction in ROS and increase in antioxidant capacity; e.g. the improvement in SOD and GST activity could potentially be sufficient to prevent LPO.	[5]
Vascular	Apelin is a potent inotropic substance and strong vasodilator, ET-1 is well-known vasoconstrictor that favors the accumulation of fibrosis by activating myofibroblasts, and it has been implicated in the modulation of cardiac function	Lower levels of ET-1 mRNA in LV and RV, and higher RV apelin can improve myocardial contractility.	[19]
GSK-3β signaling	GSK-3β is an enzyme recognized to be involved in many cell processes. The inhibition of GSK-3β *via* phosphorylation, enhances VEGF activation, calcineurin/ NFAT, or mTOR, and promotes hypertrophy, vasculogenesis and angiogenesis.	Decrease in phosphorylated GSK-3β / GSK-3 ratio promotes reduction in adverse remodeling in RV and pulmonary artery.	[20]
Neurohumoral modulation	Promotion of collagen expression and RV fibrosis by signaling through the Fn14-RhoA-MAL axis and prevention of VEGF mRNA down-regulation		[10, 21]

**Table 2:** A summary of the major outcomes from preclinical and clinical studies on exercise training regimens for subjects with PH.

Training programs in experimental studies on rodent models of PH	Exercise rehabilitation programs for patients with PH*
Influence of PH on exercise capacity	
Despite a wide spectrum of study protocols to measure exercise endurance in existing PH animal models, there is a significant correlation between worsening of exercise-related parameters and PH development, manifested by alterations in hemodynamic and remodeling parameters	RV function is a key determinant of exercise capacity in patients with PH; 6MWT is the exercise test most widely used for comprehensive prognostic evaluation and risk assessment
Exposure to a variety of potential medical agents for PH normalized exercise capacity in rodent animal models	Current guidelines adopt a threshold of >440 m as a treatment goal for patients receiving PAH-specific drug therapy
Training program settings for PH subjects	
Aerobic exercise training: running on a treadmill, or running wheel offered for voluntary exercising	Inpatient, outpatient or home-based settings: supervised aerobic (bicycle, treadmill), along with resistance training, and/or respiratory muscle training
Exercise-based training programs differed in frequency, duration and intensity; e.g. treadmill: 30 to 60 min., 5 days a week, running speed of 10–30 m/ min., incline between 15 and 35°, progressively increased speed (2–5 meters/minute). Experimental period–up to 8 wks.	Exercise-based training programs differed in frequency, duration and intensity. They were programmed usually for 3–7 days a week. The intensity was adjusted daily to individual strengths and limitations, such as physical exertion, peak heart rate and oxygen saturation. Experimental period–up to 19 wks.
Termination of exercise: until fatigue, or exhaustion (inability to keep pace with the treadmill). Exhaustion could be established when the animal was unable to keep pace with the treadmill, even after repeated application of aversive electric stimuli	Criteria for termination of exercise could include parameters as above
Advantages of training programs adopted to subjects with PH	
The potential benefits were assessed in animals with PH exposed to chronic training programs *vs*. their sedentary counterparts with PH	The potential benefits were assessed in patients receiving rehabilitation training program *vs*. control group (e.g., usual care, routine daily activities) or in pre-post exercise comparisons
Improvement in exercise intolerance measured as time to exhaustion, distance, VO_2_max, or time to VO_2_max	Beneficial impact on 6MWD and peak exercise capacity determined from CPET
Attenuation in pulmonary arteries remodeling	Improvement in pulmonary hemodynamics with a lowering of mean or resting systolic pulmonary artery pressure
Prevention of excessive body weight loss in MCT-treated animals	Improvement in outcomes for physical component scores (QoL)
Improved survival rate (MCT-induced PH)	Lack of data about long-term effects of exercise rehabilitation on PH, including clinical worsening and survival
Major determinants of study outcomes and source of potential heterogeneity	
Both early and late exercise training programs have a beneficial impact on animal performance, but the former can display more promising results according to normalization of RV function and hypertrophy	The subgroup analyses did not reveal significant improvement in 6MWD or VO_2_max after 12–15 weeks *vs*. 3-weeks of exercise training regimen.
The resultant benefits of exercise protocols can be influenced by the method of pulmonary induction. Animals exposed to chronic hypoxia can display better results than MCT-treated subjects	Further studies are needed to perform subgroup analyses according to clinical classification of PH, severity of the disease, or settings of exercise rehabilitation program (modality, intensity, length of training program, inpatient *vs*. outpatient).
Exercise training programs can demonstrate better results with regard to recovery in exercise endurance and pulmonary vasculature and function when they include inclines and progressively increasing speed, and when training intensity is individually adjusted to VO_2_max	
Any standardization of exercise protocols would help establish appropriate animal model for PH studies	Additional trials are needed to define the optimal exercise training strategy for PH patients

* based on [1, 22]

### Limitations

There are several potential weaknesses to the present study that should be mentioned. First, the examined studies used different measures of exercise capacity; as such, further studies are needed to evaluate the effects of chronic training on the increase in VO2max. Next, particular protocols varied according to details of animal familiarization with exercise equipment; some protocols did not specify any details concerning sessions of such adaptation. Therefore further comparative studies are required to evaluate the specific conditions that can trigger the effects of chronic training programs instead of just education. Moreover, due to the small number of experiments that included voluntary exercise training, it was not possible to analyze their effects on PH or exercise endurance as compared to the forced ones, e.g. treadmill. Finally, as evidenced in risk of bias assessment–animal research was poorly reported, and often essential details regarding the used methodology were not provided by the authors, as stated in the previous literature [4, 23]. Consequently, for most parameters, it was not possible to assess the risk of bias reliably, and the results of this review must be interpreted with caution.

## Conclusion

Any standardization of exercise protocols would help establish appropriate exercise protocols for PH studies. It can be considered that:

- Despite a wide spectrum of study protocols to measure exercise endurance in animals with PH, there is a significant correlation between worsening of exercise-related parameters and PH development, manifested by alterations in haemodynamic and remodeling parameters.
- The training program improves exercise intolerance in an animal model of PH, as compared to sedentary schedule, and this effect can be accompanied with less pronounced changes in PA remodeling and better animal survival.
- Both early and late exercise training programs have a beneficial impact on animal performance, but they can differ according to the final outcomes. The former can display more promising results according to normalization of RV function and hypertrophy, and the achievement of better results in the exercise test.
- The resultant benefits of exercise protocols in training and sedentary animals can be influenced by the method of pulmonary induction. PH induction by chronic hypoxia can display better results than MCT, but further studies on this relationship, including the mechanistic implications are required.
- Exercise training programs can demonstrate better results with regard to recovery in exercise endurance and pulmonary vasculature and function when they include inclines and progressively increasing speed, and when training intensity is individually adjusted to VO_2_max.
- The process of familiarization of animals with the procedure of exercise test should be planned carefully due to a risk of its influence on the obtained results (less disease progress) in case of e.g., longer durations and higher intensifies.

## Supporting information

S1 Table Study characteristics report–preclinical experiments. *–concerns protocols with chronic exercise training; Cav 1 –Caveolin 1; CH–Chronic Hypoxia; FHR–Fawn Hooded Rat; MCT–Monocrotaline; m–meters; min–minutes; OVX–Ovariectomy; PAB–Pulmonary Artery Banding; VO_2_max–Maximal Oxygen Uptake.
(DOC)

S2 Table Characteristics of preclinical studies that reported impact of PH induction on the animal capacity and training. *–concerns protocols with chronic exercise training; #–at least one session in order to familiarize with the equipment; CEXT–continuous exercise training; HIIT–high intensity interval training; nd–no data were available; TEST–assessment of exercise capacity due to PH development; TRAINING–chronic exercise training for PH management and optionally, assessment of resultant exercise capacity.
(DOC)

S3 Table Detailed results of comparative analyses according to a variety of factors associate with training schedules and assessment of animal exercise capacity. The resultant efficacy was expressed as alterations in hemodynamic and hypertrophic parameters, taken separately. A statistically significant Q measure (P<0.05) indicates heterogeneity among two or more analyzed subgroups.
(DOC)

S4 Table Detailed results of analyses based on a variety of factors associate with training schedules and assessment of animal exercise capacity. The resultant efficacy was expressed as alterations in hemodynamic/morphologic parameters that were registered during echocardiographic measurements. The decreased response ratio (R) values reveal worsening of PH-related parameters (n = 743 animals). The analyzed parameters included: PAT, PAAT, TAPSE, AT/ET or CO.
(DOC)

S5 Table Results of body weight (BW) changes due to PH and exposure to chronic exercise training. The decreased difference in means (D) reveal animal body weight loss (n = 763 animals). A statistically significant Q measure (P<0.05) indicates heterogeneity among two or more analyzed subgroups. MCT–monocrotaline; CH–chronic hypoxia.
(DOC)

S1 Fig The influence of the adaptation procedure on the resultant exercise endurance of animals with pulmonary hypertension and disease development. The overall effect was expressed as response ratio (R), according to alterations in both hemodynamic (RVSP, mPAP) and remodeling parameters (Fulton index, PA muscularization), as well as animal exercise capacity. The increased values of response ratio (R) reveal worsening of PH-related parameters and better exercise endurance. A statistically significant Q measure indicates heterogeneity among two or more analysed subgroups (*n = 1955* animals).
(DOC)

S2 Fig Tree plot (A) demonstrates a slight (P = 0.049) relationship between the animal model of pulmonary hypertension and resultant exercise capacity achieved in a test by sedentary animals. Tree-plot (B) and the annotation below show that the training program had a significant impact on the improvement of PH-related parameters according to method of PH induction. The training animals demonstrated different severities of PH-related lesions (P = 0.0004). PH prevention (or reversal) were observed for the trained animals with chronic hypoxia (P<0.0001), but were less pronounced for the MCT-based model (P = 0.036). A statistically significant Q measure (P<0.05) indicates heterogeneity among two or more analyzed subgroups.
(DOC)

S3 Fig Funnel plots showing the distribution of published study outcomes (filled squares) vs. unpublished outcomes (open circles) estimated by Trim and Fill analysis. The dashed line represents the mean and 95% CI with the added, potentially unpublished, studies and solid line represents the published studies included into meta-analysis. The vertical dashed line represents the global estimate of efficacy. Exercise endurance (sedentary animals): overall effect size (D) for Vehicle -70.75 (-75.24–(-66.26)) vs. -2.75 (-7.10–1.61)– 54 potentially missing studies were added (A); overall effect size (R) for Vehicle 0.52 (0.48–0.56)– 0 missing studies (B); PH-related parameters (sedentary animals): overall effect size (D) for Vehicle 1.39 (1.31–1.47) vs. 0.25 (0.15–0.34)– 64 missing studies (C); overall effect size (R) for Vehicle 1.99 (1.88–2.11)– 0 missing studies (D); Exercise endurance (training animals): effect size (D) for Vehicle -16.05 (-22.98–(-9.11)) vs. -9.32 (-17.45–(-1.18)–one missing study (E); effect size (R) for Vehicle: 0.83 (0.78–0.89)– 0 missing studies (F); PH-related parameters (training animals) effect size (D) for Vehicle 0.23 (0.19–0.26) vs. 0.20 (0.16–0.24)– 5 missing studies (G); effect size (R) for Vehicle 1.43 (1.34–1.52)– 0 missing studies (H). Visual inspection and the results of Egger’s test suggest missing studies and publication bias.
(DOC)

S1 File Study references. (DOC)

S2 File PRISMA 2009 checklist. (DOC)
