# Supplementary material for: Training programs in preclinical studies. The example of pulmonary hypertension. Systematic review and meta-analysis
Source: PLoS One. 2022 Nov 15;17(11):e0276875. doi: 10.1371/journal.pone.0276875 (PMC9665399; doi:10.1371/journal.pone.0276875)
Supplement: S1 Table — *–concerns protocols with chronic exercise training; Cav 1 –Caveolin 1; CH–Chronic Hypoxia; FHR–Fawn Hooded Rat; MCT–Monocrotaline; m–meters; min–minutes; OVX–Ovariectomy; PAB–Pulmonary Artery Banding; VO2max–Maximal Oxygen Uptake. (DOC) [file pone.0276875.s001.doc]

**S1 Table. Study characteristics report.**

| **Author** | **Year** | **Species** | **Race** | **PH animal model** | **Comparator** | **Exercise test (training)** | **Exercise parameter** | **Days**  **for exercise*** | **Experimental period (total no of days)** |
| --- | --- | --- | --- | --- | --- | --- | --- | --- | --- |
| Adão R | 2018 | Rat | Wistar | MCT | Healthy subject | Treadmill running | Distance (m)  VO_2_max (ml/min kg) |  | 13; 23 |
| Alencar AK | 2017 | Rat | Wistar | MCT | Healthy subject | Treadmill running | Time to exhaustion (min) |  | 28 |
| Alencar AK | 2014 | Rat | Wistar | MCT | Healthy subject | Treadmill running | Distance (m/kg) |  | 14; 28 |
| Alencar AKN | 2018 | Rat | Wistar | MCT+OVX | Healthy subject | Treadmill running | Time to exhaustion (min) |  | 35 |
| Becker CU | 2020 | Rat | Wistar | MCT | Sedentary (healthy or with PH) | Treadmill running |  | 28 | 28 |
| Boehm M | 2019 | Mouse | C57BL/6 | PAB | Healthy subject | Treadmill running | Time to exhaustion (min) |  | 35 |
| Bogaard HJ | 2010 | Rat | Sprague-Dawley | Su5416 +CH | Healthy subject | Treadmill running | Time to exhaustion (min) |  | 49 |
| Borgdorff MA | 2015 | Rat | Wistar | PAB | Healthy subject | Running wheel | Distance (m) |  | 35 |
| Brown MB | 2017 | Rat | Sprague-Dawley | MCT | Sedentary (healthy or with PH) | Treadmill running |  | 42 | 56 |
| Colombo R | 2013 | Rat | Wistar | MCT | Sedentary (healthy or with PH) | Treadmill running |  | 21 | 21 |
| Colombo R | 2016 | Rat | Wistar | MCT | Sedentary (healthy or with PH) | Treadmill running |  | 21 | 21 |
| Courboulin A | 2012 | Rat | Sprague-Dawley | MCT | Healthy subject | Treadmill running | Distance (m) |  | 28 |
| Dromparis P | 2013 | Mouse  Rat | C57BL/6  Sprague-Dawley | CH  MCT | Healthy subject | Treadmill running | Distance (m) |  | 28; 35 |
| Enache I | 2017 | Rat | Wistar | MCT | Sedentary (healthy or with PH) | Treadmill running | % max speed (cm/sec) | 28; 42 | 28; 42; 56 |
| Fang YH | 2012 | Rat | Sprague-Dawley | PAB | Healthy subject | Treadmill running | Distance (m) |  | 28; 56 |
| Favret F | 2006 | Rat | Sprague-Dawley | CH | Sedentary (healthy or with PH) | Treadmill running |  | 31 | 31 |
| Favret F | 2001 | Rat | Sprague-Dawley | CH | Healthy subject | Treadmill running | VO_2_max (ml/kg min) |  | 21 |
| Ferraz AP. | 2021 | Rat | Wistar | MCT | Healthy subject | Treadmill running | Distance (m)  Time to exhaustion (min) | 28 | 28 |
| Frump AL | 2015 | Rat | Sprague-Dawley | Su5416 +CH+OVX  Su5416 +CH | Healthy subject | Treadmill running | VO_2_max (ml/kg h) |  | 49 |
| Gomez-Arroyo J | 2015 | Rat | Sprague-Dawley | Su5416 +CH | Healthy subject | Treadmill running | Time to exhaustion (min) |  | 28 |
| Handoko ML | 2009 | Rat | Wistar | MCT | Sedentary (healthy or with PH) | Treadmill running | Time to exhaustion (min) | 28 | 42 |
| Hargett LA | 2015 | Rat | Sprague-Dawley | Su5416 +CH+NX | Sedentary (healthy or with PH) | Treadmill running |  | 72 | 91 |
| Henderson KK | 2001 | Rat | Sprague-Dawley | CH | Sedentary (healthy or with PH) | Treadmill running | VO_2_max (ml/kg min) | 70 | 70 |
| Hu J | 2017 | Mouse | C57BL/6 | PAB | Healthy subject | Swimming | Time to exhaustion (min) |  | 21 |
| Ishii R | 2020 | Rat | Sprague-  Dawley | MCT  Su5416 +CH  PAB | Healthy subject | Treadmill running | Distance (m) | 21 | 21 |
| Kashimura O | 1991 | Rat | Wistar | CH | Sedentary (healthy or with PH) | Treadmill running | Exercise capacity (m/min) | 28; 42 | 28; 42 |
| Keserü B. | 2010 | Mouse | C57BL/6 | soluble epoxide hydrolase (sEH)-/- and CH | Healthy subject | Running wheel | Distance (m) |  | 21 |
| Kikuchi N | 2018 | Rat | Sprague-Dawley | Su5416 +CH | Healthy subject | Treadmill running | Distance (m) |  | 28 |
| Koyama M | 2014 | Mouse | C57BL6/6J | CH | Healthy subject | Treadmill running | Distance (m) |  | 28 |
| Lahm T | 2016 | Rat | Sprague-Dawley | Su5416 +CH+OVX  Su5416 +CH | Healthy subject | Treadmill running | Time to VO_2_max (min) |  | 49 |
| Marsboom G | 2012 | Rat | Sprague-Dawley | CoCl_2_  CH  MCT | Healthy subject | Treadmill running | Distance (m) |  | 28 |
| McCullough DJ | 2020 | Rat | Sprague-Dawley | Su5416 +CH | Sedentary (healthy or with PH) | Treadmill running | VO_2_max (ml/kg min) | 35 | 56 |
| Megalou AJ | 2010 | Rat | Wistar | MCT | Healthy subject | Swimming | Time to exhaustion (min) |  | 14 |
| Megalou AJ | 2012 | Rat | Wistar | MCT | Healthy subject | Swimming | Time to exhaustion (% baseline) |  | 56 |
| Moreira-Gonçalves D | 2015 | Rat | Wistar | MCT | Sedentary (healthy or with PH) | Treadmill running | Time to exhaustion (min) | 14; 28 | 28 |
| Natali AJ | 2015 | Rat | Wistar | MCT | Sedentary (healthy or with PH) | Running wheel | Distance (m) | 24 | 24 |
| Neto-Neves EM | 2017 | Rat | Sprague-Dawley | Su5416 | Healthy subject | Treadmill running | Time to exhaustion (min)  VO_2_max (ml/kg h) |  | 21; 42 |
| Nogueira-Ferreira R | 2016 | Rat | Wistar | MCT | Sedentary (healthy or with PH) | Treadmill running |  | 28 | 56 |
| Okumura K | 2015 | Rat | Sprague-Dawley | MCT | Healthy subject | Treadmill running | Distance (m) |  | 35 |
| Pacagnelli FL | 2016 | Rat | Wistar | MCT | Sedentary (healthy or with PH) | Treadmill running |  | 91 | 105 |
| Piao L | 2012 | Rat | Sprague-Dawley | PAB  MCT | Healthy subject | Treadmill running | Distance (m) |  | 28 |
| Piao L (a) | 2013 | Rat | Sprague-Dawley | Su5416 +CH  MCT | Healthy subject | Treadmill running | Distance (m) |  | 28 |
| Piao L (b) | 2013 | Rat | Sprague-Dawley | FHR | Healthy subject | Treadmill running | Distance (m) |  | 180 |
| Prins KW | 2017 | Rat | Sprague-Dawley | MCT | Healthy subject | Treadmill running | Distance (m) |  | 28 |
| Ryan JJ | 2013 | Rat | Sprague-Dawley | Su5416 +CH | Healthy subject | Treadmill running | Distance (m) |  | 21 |
| Schmidt C | 2020 | Rat | Wistar | MCT | Sedentary (healthy or with PH) | Treadmill running |  | 28 | 56 |
| Schroll S | 2010 | Rat | Wistar | BLEO | Healthy subject | Treadmill running | Exercise capacity (% baseline) |  | 14; 28 |
| Schroll S (a) | 2013 | Rat | Wistar | BLEO | Healthy subject | Treadmill running | Exercise capacity (% baseline) |  | 28 |
| Schroll S (b) | 2013 | Rat | Wistar | MCT | Healthy subject | Treadmill running | Exercise capacity (% baseline) |  | 14; 28 |
| Sengul A | 2016 | Rat | Wistar albino | MCT | Healthy subject | Swimming | Time to exhaustion (min) |  | 35 |
| Silva AF | 2019 | Rat | Wistar | MCT | Healthy subject | Treadmill running | VO_2_max (ml/ kg^0,75 min)  Time to exhaustion (min) |  | 25 |
| Silva FJJ | 2021 | Rat |  | MCT | Sedentary (healthy or with PH) | Treadmill running |  | 32 | 32 |
| Soares LL | 2019 | Rat | Wistar | MCT | Sedentary (healthy or with PH) | Running wheel |  | 28 | 28 |
| Souza-Rabbo MP | 2008 | Rat | Wistar | MCT | Sedentary (healthy or with PH) | Treadmill running |  | 21; 28; 35 | 21; 28; 35 |
| Suen CM | 2019 | Rat | Sprague-Dawley  Fisher | Su5416 +CH | Healthy subject | Treadmill running | Distance (m)  Time to exhaustion (min) |  | 31 |
| Vieira JS | 2020 | Mouse | C57BL6/J | MCT | Sedentary (healthy or with PH) | Treadmill running | Distance (m) | 30 | 60; 120; 150 |
| Weissmann N | 2014 | Mouse | C57BL/6J | CH | Sedentary (healthy or with PH) | Treadmill running | Distance (m) | 21 | 21 |
| Willis GR | 2020 | Mouse | FVB | Hyperoxia | Healthy subject | Treadmill running | Distance (m) | 28 | 28 |
| Wong MJ | 2016 | Rat | Sprague-Dawley | CH | Healthy subject | Treadmill running | Distance (m) |  | 21 |
| Wu J | 2019 | Rat | Sprague-Dawley | CH | Healthy subject | Treadmill running | Distance (m) |  | 28 |
| Wunderlich C | 2008 | Mouse |  | Cav-1 knockout | Healthy subject | Swimming | Time to exhaustion (min) |  |  |
| Zimmer A | 2017 | Rat | Wistar | MCT | Sedentary (healthy or with PH) | Treadmill running |  | 35 | 35 |

* – concerns protocols with chronic exercise training; Cav 1 – Caveolin 1; CH – Chronic Hypoxia; FHR – Fawn Hooded Rat; MCT – Monocrotaline; m –meters; min – minutes; OVX – Ovariectomy; PAB – Pulmonary Artery Banding; VO_2_max – Maximal Oxygen Uptake
