# Supplementary material for: Training programs in preclinical studies. The example of pulmonary hypertension. Systematic review and meta-analysis
Source: PLoS One. 2022 Nov 15;17(11):e0276875. doi: 10.1371/journal.pone.0276875 (PMC9665399; doi:10.1371/journal.pone.0276875)
Supplement: S2 Table — *–concerns protocols with chronic exercise training; #–at least one session in order to familiarize with the equipment; CEXT–continuous exercise training; HIIT–high intensity interval training; nd–no data were available; TEST–assessment of exercise capacity due to PH development; TRAINING–chronic exercise training for PH management and optionally, assessment of resultant exercise capacity. (DOC) [file pone.0276875.s002.doc]

**S2 Table. Characteristics of studies that reported impact of PH induction on the animal capacity and training.**

| **Author** | **Year** | **Aim of the exercise protocol** | **Management with PH*** | **Method to assess exercise capacity*** | **Effort to overcome** | **Session time** | **The end-point measurement** | **Initial adaptation/pre-training (period)#** | **Exercise protocol for sedentary animals** |
| --- | --- | --- | --- | --- | --- | --- | --- | --- | --- |
| Adão R | 2018 | TEST |  | Treadmill running | Progressively increased speed from 10 m/min and 5 m/min every 5 minutes; 15° slope |  | 30 min. or until exhaustion | nd |  |
| Alencar AK | 2017 | TEST |  | Treadmill running | Increased exercise intensity until 18 m/min. |  | Until exhaustion – loss of righting reflex | nd |  |
| Alencar AK | 2014 | TEST |  | Treadmill running | Increased exercise intensity until 18 m/min. |  | Until exhaustion – loss of righting reflex | Yes |  |
| Alencar AKN | 2018 | TEST |  | Treadmill running | Three min. at 12 m/min, with 1.2 m/min increases in speed every 3 min. |  | Until exhaustion – ceassation of running | Yes (7 days) |  |
| Boehm M | 2019 | TEST |  | Treadmill running | No detailed data | | | | |
| Bogaard HJ | 2010 | TEST |  | Treadmill running | Running speed of 15 m/min; 15° slope |  | Until exhaustion – animals accepted 3 consecutive electric stimulus as opposed to running | Yes |  |
| Borgdorff MA | 2015 | TEST |  | Running wheel | Free access to a running wheel; running distance was recorded daily |  | nd | nd |  |
| Courboulin A | 2012 | TEST |  | Treadmill running | No detailed data |  | nd | nd |  |
| Dromparis P | 2013 | TEST |  | Treadmill running | Increased exercise intensity until 18 m/min and failure |  | Until failure >5 consecutive seconds on the shocker grid | Yes |  |
| Fang YH | 2012 | TEST |  | Treadmill running | Increased exercise intensity until 30 m/min |  | nd | nd |  |
| Favret F | 2001 | TEST |  | Treadmill running | Increased exercise intensity until VO_2_max was reached; 10° slope |  | nd | nd |  |
| Ferraz AP | 2021 | TEST |  | Treadmill running | Progressively increased speed (by 4-6 m/min) until 8 m/min | 15 min; 5 days a week | Until exhaustion – cessation of running | Yes (7 days) |  |
| Frump AL | 2015 | TEST |  | Treadmill running | Increased exercise intensity (by 5 m/min and 5° slope for each consecutive stage) |  | Until VO_2_ plateaued despite increasing workload, or until exhaustion – animals accepted 3 consecutive electric stimulus as opposed to running | Yes (14 days) |  |
| Gomez-Arroyo J | 2015 | TEST |  | Treadmill running | Running speed of 15 m/min; 5° slope |  | Until exhaustion – animals accepted 5 consecutive electric stimulus as opposed to running or ceased running | nd |  |
| Hu J | 2017 | TEST |  | Swimming | Animals were individually placed into beaker filled with water. Paper clip weighing 2 gram was attached to the tail to increase the resistance to swimming |  | Animals were allowed to swim until they appeared to be exhausted | nd |  |
| Ishii R | 2020 | TEST |  | Treadmill running | Running speed of 15 m/min; 15° slope (MCT)  Progressively increased speed (by 3 m/min) until 6 m/min; 15° slope (PAB, Su5416 +CH) | 10 min | 10 min  Until exhaustion – cessation of running | nd |  |
| Keserü B. | 2010 | TEST |  | Running wheel | No detailed data | | | | |
| Kikuchi N | 2018 | TEST |  | Treadmill running | Increased exercise intensity (by 5 m/min every 5 min) until 20-30 m/min. |  | Until exhaustion |  |  |
| Koyama M | 2014 | TEST |  | Treadmill running | Increased exercise intensity until 13 m/min; constant slope at 5° |  | Until exhaustion –spending 50% of the time in a stage or 3 consecutive seconds on the shock grid | nd |  |
| Lahm T | 2016 | TEST |  | Treadmill running | After 5 min-warm up, 45 min of running at the speed corresponding to 75% of VO_2_ reserve; 10° slope |  | nd | Yes (7 days) |  |
| Marsboom G | 2012 | TEST |  | Treadmill running | No detailed data |  | Until exhaustion |  |  |
| Megalou AJ | 2010 | TEST |  | Swimming | The animals were placed in a cylinder beaker (height: 50 cm; diameter: 30 cm) filled with water (25°C) to a height of 25 cm |  | Swimming represents a time from immersion until near-drowning | nd |  |
| Megalou AJ | 2012 | TEST |  | Swimming | The animals were placed in a cylinder beaker (height: 50 cm; diameter: 30 cm) filled with water (25°C) to a height of 25 cm |  | Swimming represents a time from immersion until near-drowning | nd |  |
| Neto-Neves EM | 2017 | TEST |  | Treadmill running | Increased exercise intensity (by 3 m/min every 3 min) until 25 m/min with increasing slope until 20° |  | Until VO_2_ plateaued despite increasing workload, or until exhaustion – animals accepted 3 consecutive electric stimulus as opposed to running | Yes (8 days) |  |
| Okumura K | 2015 | TEST |  | Treadmill running | Running speed of 10 m/min; 5° slope |  | Until exhaustion – animals accepted 3 consecutive electric stimulus as opposed to running | Yes (14 days) |  |
| Piao L | 2012 | TEST |  | Treadmill running | Progressively increased speed from 10 m/min and 5 m/min every 5 min. |  | 30 min. or until exhaustion | nd |  |
| Piao L (a) | 2013 | TEST |  | Treadmill running | Progressively increased speed from 10 m/min and 5 m/min every 5 min. |  | 30 min. or until exhaustion | nd |  |
| Piao L (b) | 2013 | TEST |  | Treadmill running | No detailed data | | | | |
| Prins KW | 2017 | TEST |  | Treadmill running | Progressively increased speed from 10 m/min and 5 m/min every 5 minutes |  | 30 min. or until exhaustion | nd |  |
| Ryan JJ | 2013 | TEST |  | Treadmill running | Progressively increased speed from 5 m/min and 5 m/min every 5 min. |  | 30 min. or until exhaustion | nd |  |
| Schroll S | 2010 | TEST |  | Treadmill running | The baseline: max. exercise capacity (running speed of 20 m/min; 12° slope) |  | Until exhaustion – third time the animal no longer kept pace with the speed of the treadmill | Yes (7 days) |  |
| Schroll S (a) | 2013 | TEST |  | Treadmill running | The baseline: max. exercise capacity |  |  | Yes (7 days) |  |
| Schroll S (b) | 2013 | TEST |  | Treadmill running | The baseline: max. exercise capacity (running speed of 25 m/min; 12° slope) |  | Until exhaustion – third time the animal no longer kept pace with the speed of the treadmill | Yes (7 days) |  |
| Sengul A | 2016 | TEST |  | Swimming | The animals were placed in a cylinder beaker (height: 50 cm; diameter: 30 cm) filled with water (25°C) |  | Swimming duration was calculated subtracting the motionless period from the swimming time | nd |  |
| Silva AF | 2019 | TEST |  | Treadmill running | 5 min warm-up at 15 m/min and 10° slope, followed by treadmill speed increments of 1.8 m/min every 2 min. |  | Until exhaustion – animals accepted 3 consecutive electric stimulus as opposed to running | nd |  |
| Suen CM | 2019 | TEST |  | Treadmill running | Progressively increased speed until 25 m/min. |  | Until exhaustion – animals accepted 3 consecutive electric stimulus as opposed to running | Yes (1day) |  |
| Willis GR | 2020 | TEST |  | Treadmill running | Progressively increased speed (from 3 m/min until 12 m/min) | 13 min | Until exhaustion – cessation of running | nd |  |
| Wong MJ | 2016 | TEST |  | Treadmill running | Progressively increased speed (by 2 m/min every 5 min) until 20 m/min; 10° slope |  | Until exhaustion – delayed (>2 s) righting reflex | Yes (7 days) |  |
| Wu J | 2019 | TEST |  | Treadmill running | Progressively increased speed until 18 m/min. |  | Until exhaustion – animal remained for 5s on the shock grid | Yes (21 days, once a week) |  |
| Wunderlich C | 2008 | TEST |  | Swimming | A 4-l beaker filled with water (34°C) |  | Until the animal was initially unable to maintain complete buoyancy | nd |  |
| Becker CU | 2020 | TRAINING | EARLY | Treadmill running | Training: Progressively increased speed from by 3 m/min every 3 minute 60% of the max. speed achieved (75% of VO_2_max) | 50 min; 5 days a week |  | Yes (14 days) | nd |
| Brown MB | 2017 | TRAINING | LATE | Treadmill running | Training: CEXT or HIIT | 60 or 30 min; 5 days a week | nd | Yes (20 days) | nd |
| Colombo R | 2013 | TRAINING | EARLY | Treadmill running | Training: 60% of VO_2_max | 50-60 min; 5 days a week | nd | Yes (14 days) | nd |
| Colombo R | 2016 | TRAINING | EARLY | Treadmill running | Training: 60% of VO_2_max | 60 min; 5 days a week | nd | Yes (14 days) | nd |
| Enache I | 2017 | TRAINING | LATE | Treadmill running | Training: 65% of VO_2_max reached by each rat at 9 m/min; (−15°) slope | 30 min; 5 days a week | nd | Yes (14 days) | Run on the downhill treadmill 3 times a week for 5 min; (−15°) slope |
| Favret F | 2006 | TRAINING | EARLY | Treadmill running | Training: running speed from 30 m/min and was increased; 10° slope; test: increased exercise intensity until VO_2_ max was reached | 60 min; 5 days a week | nd | Yes | nd |
| Handoko ML | 2009 | TRAINING | LATE | Treadmill running | Training: running speed of 13.3 m/min; no slope. test: running speed of 15 m/min; 20° slope | 30 min; 5 days a week | Until exhaustion – animals accepted 3 consecutive electric stimulus as opposed to running. Maximal running time was 90 min. | Yes (14 days) | Run on the treadmill 5 times a week for 1 min at 13.3 m/min; no slope |
| Hargett LA | 2015 | TRAINING | LATE | Treadmill running | Animals selected for the exercise study on the basis of willingness to run (mild electrical stimulation).Training: started at 80% of the VO_2_ max of sedentary subjects and increased exercise intensity; 5° slope | 60 min; 5 days a week | nd | Yes (7 days) | nd |
| Henderson KK | 2001 | TRAINING | EARLY | Treadmill running | Training: started at 80% of the VO_2_ max of normoxic sedentary animals with increased intensity until 30 m/min; 10° slope. Test: increased exercise intensity until VO_2_ max was reached | 60 min; 5 days a week | nd | nd | nd |
| Kashimura O | 1991 | TRAINING | EARLY | Treadmill running | Training: increased exercise intensity until 32 m/min; 3−5° slope | 20-40 min; 6 days a week | nd | Yes (6 days) | nd |
| McCullough DJ | 2020 | TRAINING | LATE | Treadmill running | Training: running speed of 15 m/min; 15° slope (55–60% relative VO_2_max of the sedentary subjects); test: VO_2_max; increasing speed and/or incline every 3–5 min. | 60 min; 5 days a week | nd | Yes (7 days) | nd |
| Moreira-Gonçalves D | 2015 | TRAINING | EARLY; LATE | Treadmill running | Training: constant running speed of 25-30 m/min; test: constant speed of 20 m/min. | 60 min; 5 days a week | Until exhaustion – animals accepted 3 consecutive electric stimulus as opposed to running. Maximal running time was 110 min.(test) | Yes (7 days) | Placed on the treadmill 3 times a week for 5 min without any movement, followed by 1 min at a running speed of 10 m/min. |
| Natali AJ | 2015 | TRAINING | EARLY | Running wheel | Training: free access to a stainless steel vertical running wheel; running distance was recorded daily |  |  | Yes (2 days) | nd |
| Nogueira-Ferreira R | 2016 | TRAINING | EARLY | Treadmill running | Training: increased exercise intensity until 25 m/min. | 60 min; 5 days a week | nd | nd | Placed on a non-moving treadmill 3 times a week for 5 min without any movement |
| Pacagnelli FL | 2016 | TRAINING | EARLY | Treadmill running | Training: increased exercise intensity until 10-15 m/min. | 60 min; 5 days a week | nd | Yes (14 days) | nd |
| Schmidt C | 2020 | TRAINING | EARLY | Treadmill running | Training: progressively increased speed until 25 m/min. | 60 min; 5 days a week | nd | Yes (7 days) | Placed on the treadmill 3 times /week, for 10 min, without movement |
| Silva FJJ | 2021 | TRAINING | LATE | Treadmill running | Training: 60% of VO_2_max achieved until 60 min | 60 min | Until exhaustion – cessation of running | nd | nd |
| Soares LL | 2019 | TRAINING | EARLY | Running wheel | Training: The exercised subjects were haused individually and had free access to the running wheel. Test: Progressively increased speed from 5 m/min and 3 m/min every 3 min. |  | Until exhaustion – animal no longer kept pace with the speed of the treadmill and remained for 10s on the shock grid at the back of the treadmill rather than run | Yes (3 days) | nd |
| Souza-Rabbo MP | 2008 | TRAINING | EARLY | Treadmill running | Training: running speed of 10–15 m/min. | 50-60 min; 5 days a week | nd | Yes (14 days) | nd |
| Vieira JS | 2020 | TRAINING | LATE | Treadmill running | Training: 60% of the max. speed achieved in the progressive exercise test; test: increased exercise intensity by 3 m/min every 3 min. | 60 min; 5 days a week | Until exhaustion – cessation of running | Yes (7 days) | nd |
| Weissmann N | 2014 | TRAINING | EARLY | Treadmill running | Training: 60% of the max. speed achieved in the progressive exercise test; test: increased exercise intensity by 3 m/min every 3 min; 25°slope | 60 min; 5 days a week | Until exhaustion – cessation of running | Yes (7 days) | nd |
| Zimmer A | 2017 | TRAINING | EARLY | Treadmill running | Training: 60% of VO_2_max achieved in the progressive exercise test (by 0.3 m/min every 3 min) | 60 min | nd | Yes (14 days) | nd |

* – concerns protocols with chronic exercise training; # – at least one session in order to familiarize with the equipment; CEXT – continuous exercise training; HIIT – high intensity interval training; nd – no data were available; TEST – assessment of exercise capacity due to PH development; TRAINING – chronic exercise training for PH management and optionally, assessment of resultant exercise capacity.
