# Supplementary material for: Training programs in preclinical studies. The example of pulmonary hypertension. Systematic review and meta-analysis
Source: PLoS One. 2022 Nov 15;17(11):e0276875. doi: 10.1371/journal.pone.0276875 (PMC9665399; doi:10.1371/journal.pone.0276875)
Supplement: S3 Table — The resultant efficacy was expressed as alterations in hemodynamic and hypertrophic parameters, taken separately. A statistically significant Q measure (P<0.05) indicates heterogeneity among two or more analyzed subgroups. (DOC) [file pone.0276875.s003.doc]

**S3 Table. Detailed results of comparative analyses according to a variety of factors according to training schedules and assessment of animal exercise capacity.**

The resultant efficacy was expressed as alterations in hemodynamic and hypertrophic parameters, taken separately. AR – artery remodeling; HEM – haemodynamic parameter; RVH – right ventricle hypertrophy.

| **Reference** | **Item** | **R (95% CI)** | **Comparative analysis** | **Parameter** |
| --- | --- | --- | --- | --- |
| **Fig. 3A** | Metaregression | log R=0.93−0.48*exercise | P=0.02 | HEM |
|  |  | log R=0.95−0.54*exercise | P=0.038 | AR |
|  |  | log R=0.96−0.58*exercise | P=0.002 | RVH |
| **Fig. 4D** | Adaptation – YES | 2.06 (1.79−2.37); P<0.0001 | Q=0.013; df=1; P>0.05 | HEM |
|  | Adaptation – NO | 2.09 (1.84−2.35); P<0.0001 |  |  |
|  | Adaptaion – YES | 1.66 (1.50−1.84); P<0.0001 | Q=8.56; df=1; P=0.003 | AR |
|  | Adaptation – NO | 2.14 (1.87−2.45); P<0.0001 |  |  |
|  | Adaptaion – YES | 1.91 (1.67−2.19); P<0.0001 | Q=0.25; df=1; P>0.05 | RVH |
|  | Adaptation – NO | 2.00 (1.77−2.26); P<0.0001 |  |  |
| **Fig. 5E** | SEDENTARY GROUP | 1.60 (1.42−1.80); P<0.0001 | Q=0.85; df=1; P>0.05 | HEM |
|  | TRAINING GROUP | 1.41 (1.11−1.80); P<0.0001 |  |  |
|  | SEDENTARY GROUP | 1.84 (1.46−2.32); P<0.0001 | Q=4.51; df=1; P=0.03 | AR |
|  | TRAINING GROUP | 1.37 (1.20−1.57); P<0.0001 |  |  |
|  | SEDENTARY GROUP | 1.54 (1.36−1.76); P<0.0001 | Q=0.72; df=1; P>0.05 | RVH |
|  | TRAINING GROUP | 1.45 (1.36−1.56); P<0.0001 |  |  |
| **Fig. 5F** | EARLY TRAINING | 1.24 (1.11−1.38); P=0.02 | Q=2.0; df=1; P>0.05 | HEM |
|  | LATE TRAINING | 1.54 (1.06−2.26); P<0.0001 |  |  |
|  | EARLY TRAINING | 1.27 (1.20−1.36); P<0.0001 | Q=7.53; df=1; P=0.006 | RVH |
|  | LATE TRAINING | 1.69 (1.40−2.04); P<0.0001 |  |  |
