# Supplementary material for: Training programs in preclinical studies. The example of pulmonary hypertension. Systematic review and meta-analysis
Source: PLoS One. 2022 Nov 15;17(11):e0276875. doi: 10.1371/journal.pone.0276875 (PMC9665399; doi:10.1371/journal.pone.0276875)
Supplement: S4 Table — The resultant efficacy was expressed as alterations in hemodynamic/morphologic parameters that were registered during echocardiographic measurements. The decreased response ratio (R) values reveal worsening of PH-related parameters (n = 743 animals). The analyzed parameters included: PAT, PAAT, TAPSE, AT/ET or CO. (DOC) [file pone.0276875.s004.doc]

**S4 Table**. **Detailed results of analyses based on a variety of factors associate with training schedules and assessment of animal exercise capacity.**

| **Item** | **D (95% CI)** | **Comparative analysis** | **Comment** |
| --- | --- | --- | --- |
| Sedentary animals | -41.08 (-60.55−(-21.62)); P<0.0001 | Q= 1.59; df=1; P>0.05 | Overall effect of chronic exercise training on BW |
| Training animals | -26.97 (-37.01−(-16.94)); P<0.0001 |
| Early training program | -24.09 (-31.5−(-16.67)); P<0.0001 | Q=0.91; df=1; P>0.05 | Overall effect of chronic exercise training on BW – training subgroup |
| Late training program | -38.30 (-66.60−(-10.01)); P=0.008 |
| Sedentary animals | -49.36 (-56.55−(-42.17)); P<0.0001 | Q=7.83; df=1; P=0.005 | Overall effect of chronic exercise training on BW – MCT subgroup |
| Training animals | -27.41 (-41.00−(-13.82)); P<0.0001 |
| Sedentary animals | -14.36 (-39.59−10.87)); P>0.05 | Q=0.42; df=1; P>0.05 | Overall effect of chronic exercise training on BW – CH subgroup |
| Training animals | -24.41 (-40.76−(-8.06)); P=0.003 |

The resultant efficacy was expressed as alterations in hemodynamic/morphologic parameters that were registered during echacardiographic measurements. The decreased response ratio (R) values reveal worsening of PH-related parameters *(n= 743 animals)*. The analyzed parameters included: PAT, PAAT, TAPSE, AT/ET or CO.
