# Supplementary material for: Training programs in preclinical studies. The example of pulmonary hypertension. Systematic review and meta-analysis
Source: PLoS One. 2022 Nov 15;17(11):e0276875. doi: 10.1371/journal.pone.0276875 (PMC9665399; doi:10.1371/journal.pone.0276875)
Supplement: S5 Table — The decreased difference in means (D) reveal animal body weight loss (n = 763 animals). A statistically significant Q measure (P<0.05) indicates heterogeneity among two or more analyzed subgroups. MCT–monocrotaline; CH–chronic hypoxia. (DOC) [file pone.0276875.s005.doc]

**S5 Table**. **Results of body weight (BW) changes due to PH and exposure to chronic exercise training.**

| **Item** | **R (95% CI)** | **Comparative analysis** | **Comment** |
| --- | --- | --- | --- |
| Overall effect of PH Induction | 0.63 (0.57−0.70); P<0.0001 | - | Sedentary subgroup |
| Early training program | 0.94 (0.80−1.11); P>0.05 | Q=7.62; df=1; P=0.006 | Overall effect of chronic exercise training on PH according to the schedule |
| Late training program | 0.69 (0.59−0.81); P<0.0001 |
| Treadmill running | 0.77 (0.71−0.84); P<0.0001 | Q=0.84; df=2; P>0.05 | Overall effect of chronic exercise training on PH according to the method |
| Running wheel | 0.82 (0.74−0.91); P=0.0002 |

The decreased difference in means (D) reveal animal body weight loss *(n= 763 animals)*; MCT – monocrotaline; CH – chronic hypoxia.
