# Supplementary material for: Training programs in preclinical studies. The example of pulmonary hypertension. Systematic review and meta-analysis
Source: PLoS One. 2022 Nov 15;17(11):e0276875. doi: 10.1371/journal.pone.0276875 (PMC9665399; doi:10.1371/journal.pone.0276875)
Supplement: S1 Fig — The overall effect was expressed as response ratio (R), according to alterations in both hemodynamic (RVSP, mPAP) and remodeling parameters (Fulton index, PA muscularization), as well as animal exercise capacity. The increased values of response ratio (R) reveal worsening of PH-related parameters and better exercise endurance. A statistically significant Q measure indicates heterogeneity among two or more analysed subgroups (n = 1955 animals). (DOC) [file pone.0276875.s006.doc]

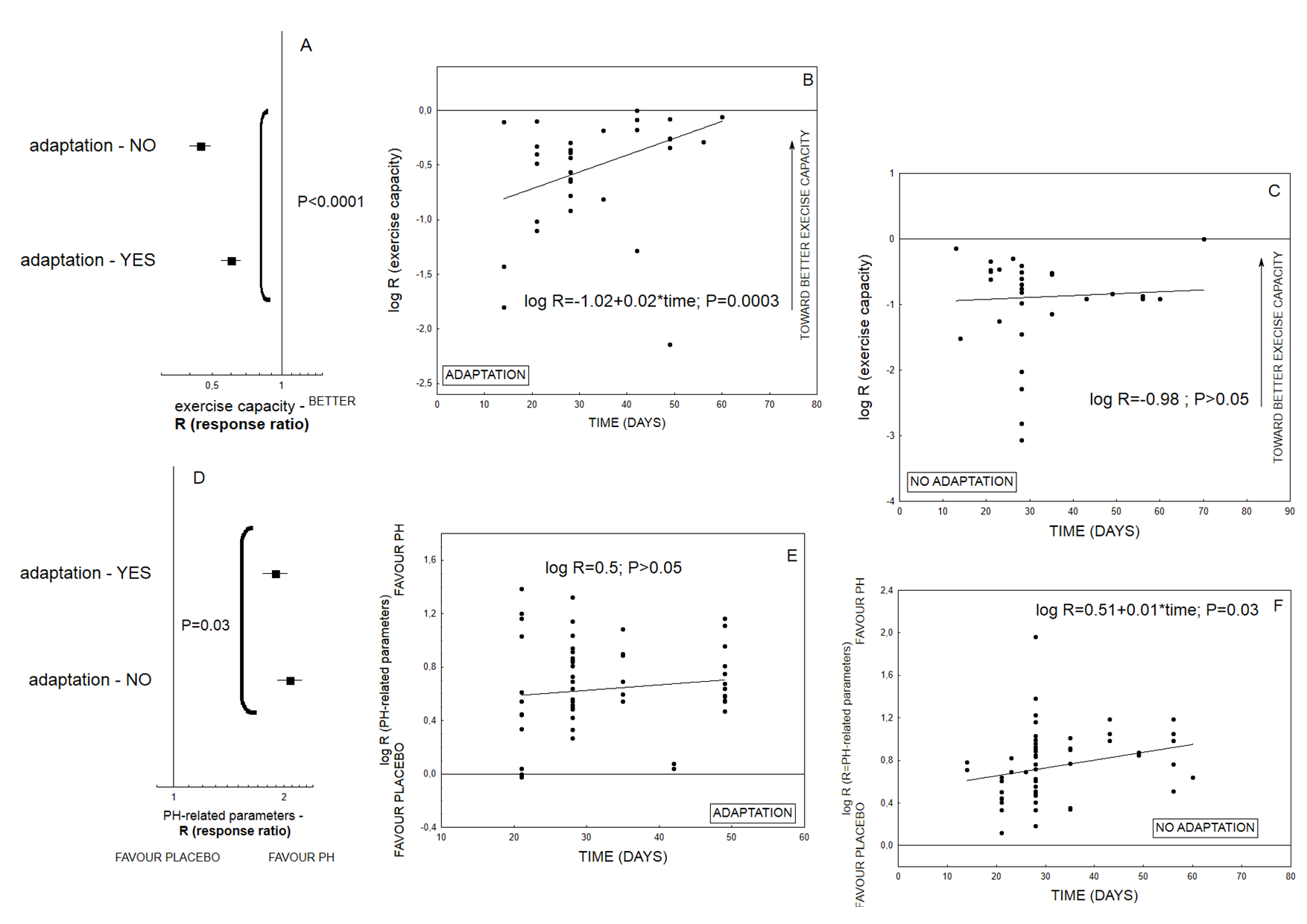
**S1 Fig.** The influence of the adaptation procedure on the resultant exercise endurance of animals with pulmonary hypertension and disease development. The overall effect was expressed as response ratio (R), according to alterations in both hemodynamic (RVSP, mPAP) and remodeling parameters (Fulton index, PA muscularization), as well as animal exercise capacity. The increased values of response ratio (R) reveal worsening of PH-related parameters and better exercise endurance. A statistically significant Q measure indicates heterogeneity among two or more analysed subgroups (n= 1955 animals)
