# Supplementary material for: Training programs in preclinical studies. The example of pulmonary hypertension. Systematic review and meta-analysis
Source: PLoS One. 2022 Nov 15;17(11):e0276875. doi: 10.1371/journal.pone.0276875 (PMC9665399; doi:10.1371/journal.pone.0276875)
Supplement: S2 Fig — Tree plot (A) demonstrates a slight (P = 0.049) relationship between the animal model of pulmonary hypertension and resultant exercise capacity achieved in a test by sedentary animals. Tree-plot (B) and the annotation below show that the training program had a significant impact on the improvement of PH-related parameters according to method of PH induction. The training animals demonstrated different severities of PH-related lesions (P = 0.0004). PH prevention (or reversal) were observed for the trained animals with chronic hypoxia (P<0.0001), but were less pronounced for the MCT-based model (P = 0.036). A statistically significant Q measure (P<0.05) indicates heterogeneity among two or more analyzed subgroups. (DOC) [file pone.0276875.s007.doc]

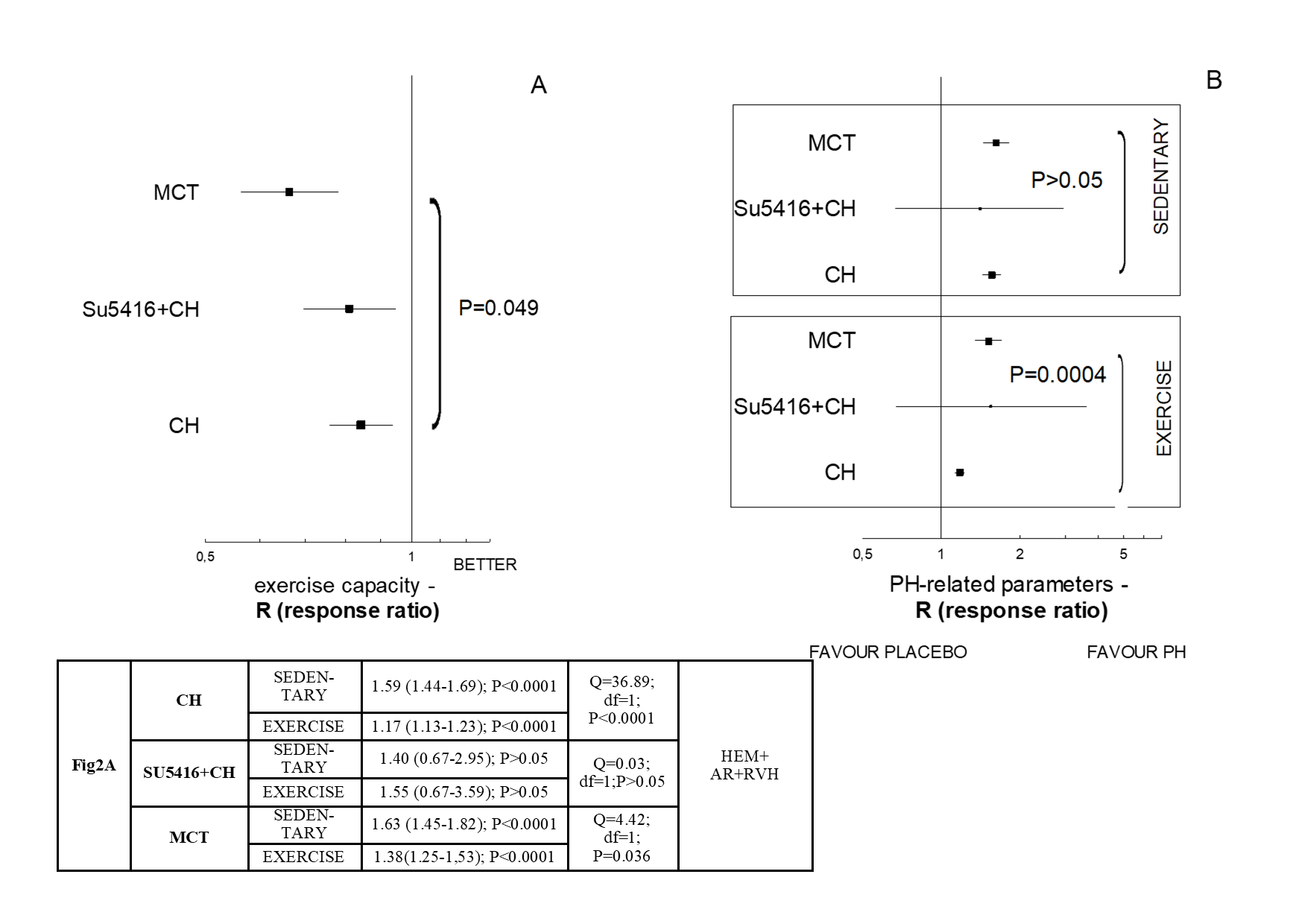
**S2 Fig.** Tree plot (**A**) demonstrates a slight (P=0.049) relationship between the animal model of pulmonary hypertension and resultant exercise capacity achieved in a test by sedentary animals. Tree-plot (**B**) and the annotation below show that the training program had a significant impact on the improvement of PH-related parameters according to method of PH induction. The training animals demonstrated different severities of PH-related lesions (P=0.0004). PH prevention (or reversal) were observed for the trained animals with chronic hypoxia (P<0.0001), but were less pronounced for the MCT-based model (P=0.036). AR – artery remodeling; HEM – haemodynamic parameter; RVH – right ventricle hypertrophy.
