# Supplementary material for: Training programs in preclinical studies. The example of pulmonary hypertension. Systematic review and meta-analysis
Source: PLoS One. 2022 Nov 15;17(11):e0276875. doi: 10.1371/journal.pone.0276875 (PMC9665399; doi:10.1371/journal.pone.0276875)
Supplement: S3 Fig — The dashed line represents the mean and 95% CI with the added, potentially unpublished, studies and solid line represents the published studies included into meta-analysis. The vertical dashed line represents the global estimate of efficacy. Exercise endurance (sedentary animals): overall effect size (D) for Vehicle -70.75 (-75.24–(-66.26)) vs. -2.75 (-7.10–1.61)– 54 potentially missing studies were added (A); overall effect size (R) for Vehicle 0.52 (0.48–0.56)– 0 missing studies (B); PH-related parameters (sedentary animals): overall effect size (D) for Vehicle 1.39 (1.31–1.47) vs. 0.25 (0.15–0.34)– 64 missing studies (C); overall effect size (R) for Vehicle 1.99 (1.88–2.11)– 0 missing studies (D); Exercise endurance (training animals): effect size (D) for Vehicle -16.05 (-22.98–(-9.11)) vs. -9.32 (-17.45–(-1.18)–one missing study (E); effect size (R) for Vehicle: 0.83 (0.78–0.89)– 0 missing studies (F); PH-related parameters (training animals) effect size (D) for Vehicle 0.23 (0.19–0.26) vs. 0.20 (0.16–0.24)– 5 missing studies (G); effect size (R) for Vehicle 1.43 (1.34–1.52)– 0 missing studies (H). Visual inspection and the results of Egger’s test suggest missing studies and publication bias. (DOC) [file pone.0276875.s008.doc]

**S3 Fig.** **Funnel plots showing the distribution of published study outcomes (filled squares) vs. unpublished outcomes (open circles) estimated by Trim and Fill analysis. The dashed line represents the mean and 95% CI with the added, potentially unpublished, studies and solid line represents the published studies included into meta-analysis. The vertical dashed line represents the global estimate of efficacy.** Exercise endurance (sedentary animals): overall effect size (D) for Vehicle -70.75 (-75.24–(-66.26)) *vs.* -2.75 (-7.10–1.61) – 54 potentially missing studies were added (**A**); overall effect size (R) for Vehicle 0.52 (0.48–0.56) – 0 missing studies (**B**); PH-related parameters (sedentary animals): overall effect size (D) for Vehicle 1.39 (1.31–1.47) *vs.* 0.25 (0.15–0.34) – 64 missing studies (**C**); overall effect size (R) for Vehicle 1.99 (1.88–2.11) – 0 missing studies (**D**); Exercise endurance (training animals): effect size (D) for Vehicle -16.05 (-22.98–(-9.11)) *vs*. -9.32 (-17.45–(-1.18) – one missing study (**E**); effect size (R) for Vehicle: 0.83 (0.78–0.89) – 0 missing studies (**F**); PH-related parameters (training animals) effect size (D) for Vehicle 0.23 (0.19–0.26) *vs*. 0.20 (0.16–0.24) – 5 missing studies (**G**); effect size (R) for Vehicle 1.43 (1.34–1.52) – 0 missing studies (**H**). Visual inspection and the results of Egger’s test suggest missing studies and publication bias.

**
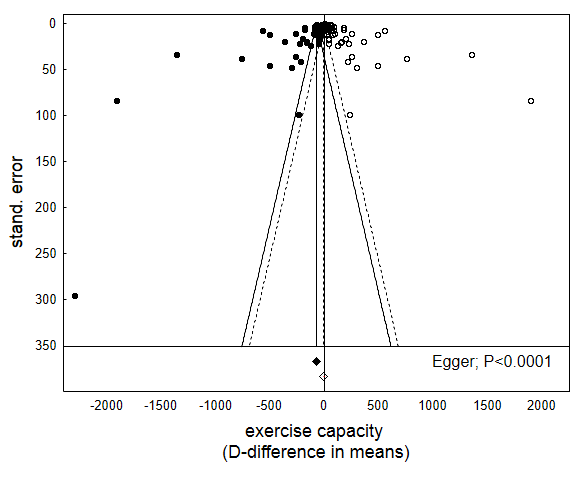

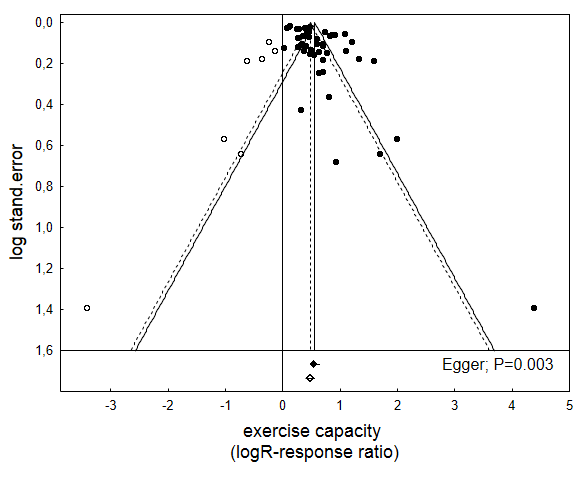
**

A

B


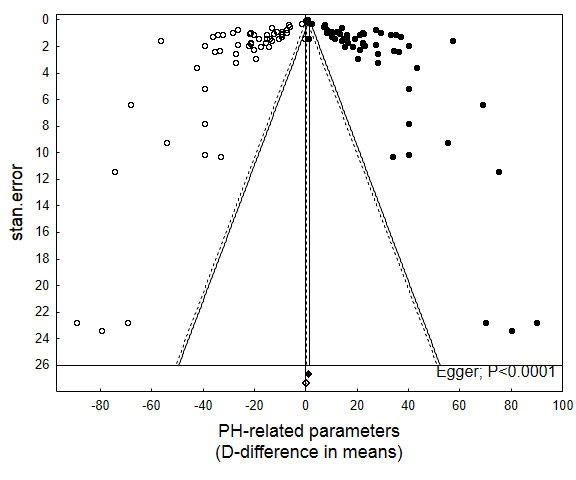

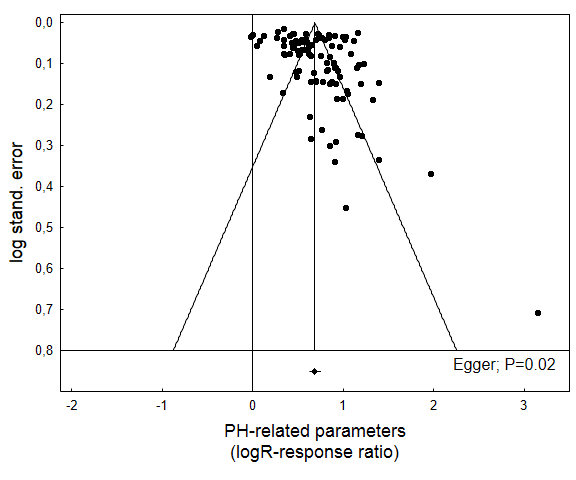


C

D


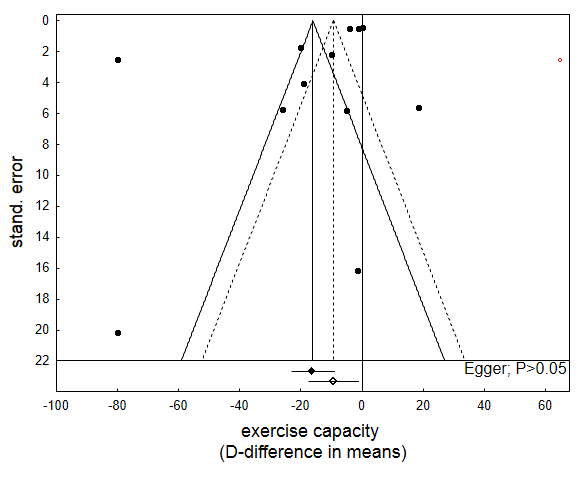

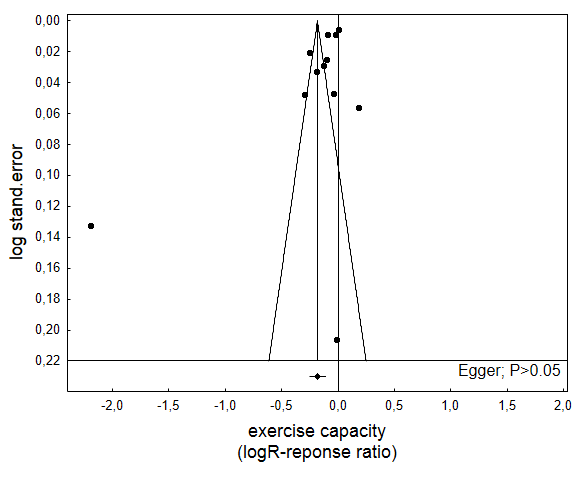


E

F


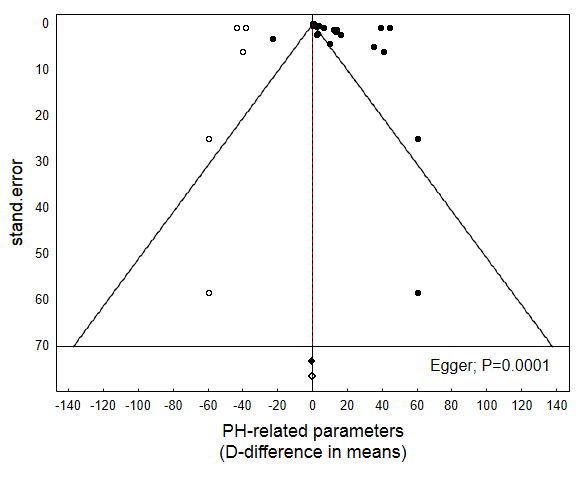

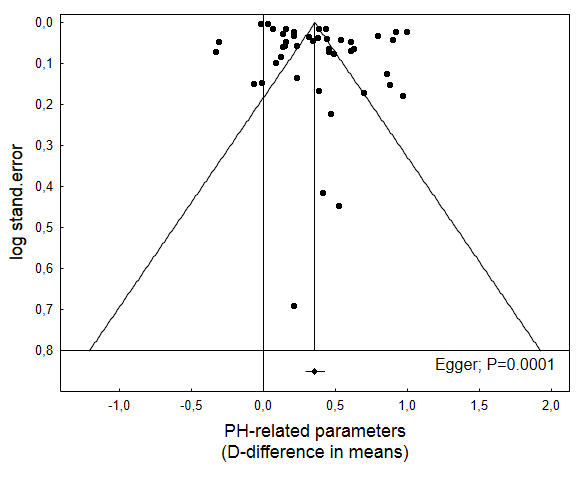


G

H
