# Supplementary material for: Training programs in preclinical studies. The example of pulmonary hypertension. Systematic review and meta-analysis
Source: PLoS One. 2022 Nov 15;17(11):e0276875. doi: 10.1371/journal.pone.0276875 (PMC9665399; doi:10.1371/journal.pone.0276875)
Supplement: S1 File — (DOC) [file pone.0276875.s009.doc]

**S1 File. Study references**

**c**

**f**

**i**

1. Adão R, Mendes-Ferreira P, Santos-Ribeiro D, Maia-Rocha C, Pimentel LD, Monteiro-Pinto C, Mulvaney EP, Reid HM, Kinsella BT, Potus F, Breuils-Bonnet S, Rademaker MT, Provencher S, Bonnet S, Leite-Moreira AF, Brás-Silva C. Urocortin-2 improves right ventricular function and attenuates pulmonary arterial hypertension. Cardiovasc Res. 2018 Jul 1;114(8):1165-1177.
2. Alencar AK, Montes GC, Montagnoli T, Silva AM, Martinez ST, Fraga AG, Wang H, Groban L, Sudo RT, Zapata-Sudo G. Activation of GPER ameliorates experimental pulmonary hypertension in male rats. Eur J Pharm Sci. 2017 Jan 15;97:208-217.
3. Alencar AK, Pereira SL, da Silva FE, Mendes LV, Cunha Vdo M, Lima LM, Montagnoli TL, Caruso-Neves C, Ferraz EB, Tesch R, Nascimento JH, Sant'anna CM, Fraga CA, Barreiro EJ, Sudo RT, Zapata-Sudo G. N-acylhydrazone derivative ameliorates monocrotaline-induced pulmonary hypertension through the modulation of adenosine AA2R activity. Int J Cardiol. 2014 May 1;173(2):154-62.
4. Alencar AKN, Montes GC, Costa DG, Mendes LVP, Silva AMS, Martinez ST, Trachez MM, Cunha VDMN, Montagnoli TL, Fraga AGM, Wang H, Groban L, Fraga CAM, Sudo RT, Zapata-Sudo G. Cardioprotection Induced by Activation of GPER in Ovariectomized Rats With Pulmonary Hypertension. J Gerontol A Biol Sci Med Sci. 2018 Aug 10;73(9):1158-1166.
5. Becker CU, Sartório CL, Campos-Carraro C, Siqueira R, Colombo R, Zimmer A, Belló-Klein A. Exercise training decreases oxidative stress in skeletal muscle of rats with pulmonary arterial hypertension. Arch Physiol Biochem. 2020 May 25:1-9.
6. Boehm M, Tian X, Mao Y, Ichimura K, Dufva MJ, Ali K, Dannewitz Prosseda S, Shi Y, Kuramoto K, Reddy S, Kheyfets VO, Metzger RJ, Spiekerkoetter E. Delineating the molecular and histological events that govern right ventricular recovery using a novel mouse model of pulmonary artery de-banding. Cardiovasc Res. 2020 Aug 1;116(10):1700-1709. doi: 10.1093/cvr/cvz310. PMID: 31738411; PMCID: PMC7643543.
7. Bogaard HJ, Natarajan R, Mizuno S, Abbate A, Chang PJ, Chau VQ, Hoke NN, Kraskauskas D, Kasper M, Salloum FN, Voelkel NF. Adrenergic receptor blockade reverses right heart remodeling and dysfunction in pulmonary hypertensive rats. Am J Respir Crit Care Med. 2010 Sep 1;182(5):652-60.
8. Borgdorff MA, Koop AM, Bloks VW, Dickinson MG, Steendijk P, Sillje HH, van Wiechen MP, Berger RM, Bartelds B. Clinical symptoms of right ventricular failure in experimental chronic pressure load are associated with progressive diastolic dysfunction. J Mol Cell Cardiol. 2015 Feb;79:244-53.
9. Brown MB, Neves E, Long G, Graber J, Gladish B, Wiseman A, Owens M, Fisher AJ, Presson RG, Petrache I, Kline J, Lahm T. High-intensity interval training, but not continuous training, reverses right ventricular hypertrophy and dysfunction in a rat model of pulmonary hypertension. Am J Physiol Regul Integr Comp Physiol. 2017 Feb 1;312(2):R197-R210.
10. Colombo R, Siqueira R, Becker CU, Fernandes TG, Pires KM, Valença SS, Souza-Rabbo MP, Araujo AS, Belló-Klein A. Effects of exercise on monocrotaline-induced changes in right heart function and pulmonary artery remodeling in rats. Can J Physiol Pharmacol. 2013 Jan;91(1):38-44.
11. Colombo R, Siqueira R, Conzatti A, de Lima Seolin BG, Fernandes TR, Godoy AE, Litvin IE, Silva JM, Tucci PJ, da Rosa Araújo AS, Belló-Klein A. Exercise training contributes to H 2 O 2/VEGF signaling in the lung of rats with monocrotaline-induced pulmonary hypertension. Vascul Pharmacol. 2016 Dec;87:49-59.
12. Courboulin A, Barrier M, Perreault T, Bonnet P, Tremblay VL, Paulin R, Tremblay E, Lambert C, Jacob MH, Bonnet SN, Provencher S, Bonnet S. Plumbagin reverses proliferation and resistance to apoptosis in experimental PAH. Eur Respir J 2012; 40: 618–629
13. Dromparis P, Paulin R, Stenson TH, Haromy A, Sutendra G, Michelakis ED. Attenuating endoplasmic reticulum stress as a novel therapeutic strategy in pulmonary hypertension. Circulation. 2013 Jan 1;127(1):115-25.
14. Enache I, Favret F, Doutreleau S, Goette Di Marco P, Charles AL, Geny B, Charloux A. Downhill exercise training in monocrotaline-injected rats: Effects on echocardiographic and haemodynamic variables and survival. Arch Cardiovasc Dis. 2017 Feb;110(2):106-115.
15. Fang YH, Piao L, Hong Z, Toth PT, Marsboom G, Bache-Wiig P, Rehman J, Archer SL. Therapeutic inhibition of fatty acid oxidation in right ventricular hypertrophy: exploiting Randle's cycle. J Mol Med (Berl). 2012 Jan;90(1):31-43.
16. Favret F, Henderson KK, Allen J, Richalet JP, Gonzalez NC. Exercise training improves lung gas exchange and attenuates acute hypoxic pulmonary hypertension but does not prevent pulmonary hypertension of prolonged hypoxia. J Appl Physiol (1985). 2006 Jan;100(1):20-5.
17. Favret F, Richalet JP, Henderson KK, Germack R, Gonzalez NC. Myocardial adrenergic and cholinergic receptor function in hypoxia: correlation with O(2) transport in exercise. Am J Physiol Regul Integr Comp Physiol. 2001 Mar;280(3):R730-8.
18. Ferraz AP, Seara FAC, Baptista EF, Barenco TS, Sottani TBB, Souza NSC, Domingos AE, Barbosa RAQ, Takiya CM, Couto MT, Resende GO, Campos de Carvalho AC, Ponte CG, Nascimento JHM. BKCa Channel Activation Attenuates the Pathophysiological Progression of Monocrotaline-Induced Pulmonary Arterial Hypertension in Wistar Rats. Cardiovasc Drugs Ther. 2021 Aug;35(4):719-732. doi: 10.1007/s10557-020-07115-5. Epub 2020 Nov 27.
19. Frump AL, Goss KN, Vayl A, Albrecht M, Fisher A, Tursunova R, Fierst J, Whitson J, Cucci AR, Brown MB, Lahm T. Estradiol improves right ventricular function in rats with severe angioproliferative pulmonary hypertension: effects of endogenous and exogenous sex hormones. Am J Physiol Lung Cell Mol Physiol. 2015 May 1;308(9):L873-90.
20. Gomez-Arroyo J, Sakagami M, Syed AA, Farkas L, Van Tassell B, Kraskauskas D, Mizuno S, Abbate A, Bogaard HJ, Byron PR, Voelkel NF. Iloprost reverses established fibrosis in experimental right ventricular failure. Eur Respir J. 2015 Feb;45(2):449-62.
21. Handoko ML, de Man FS, Happé CM, Schalij I, Musters RJ, Westerhof N, Postmus PE, Paulus WJ, van der Laarse WJ, Vonk-Noordegraaf A. Opposite effects of training in rats with stable and progressive pulmonary hypertension. Circulation. 2009 Jul 7;120(1):42-9.
22. Hargett LA, Hartman LJ, Scruggs AK, McLendon JM, Haven AK, Bauer NN. Severe pulmonary arterial hypertensive rats are tolerant to mild exercise. Pulm Circ. 2015 Jun;5(2):349-55.
23. Henderson KK, Clancy RL, Gonzalez NC. Living and training in moderate hypoxia does not improve VO2 max more than living and training in normoxia. J Appl Physiol (1985). 2001 Jun;90(6):2057-62.
24. Hu J, Sharifi-Sanjani M, Tofovic SP. Nitrite Prevents Right Ventricular Failure and Remodeling Induced by Pulmonary Artery Banding . J Cardiovasc Pharmacol. 2017 Feb;69(2):93-100.
25. Ishii R, Okumura K, Akazawa Y, Malhi M, Ebata R, Sun M, Fujioka T, Kato H, Honjo O, Kabir G, Kuebler WM, Connelly K, Maynes JT, Friedberg MK. Heart Rate Reduction Improves Right Ventricular Function and Fibrosis in Pulmonary Hypertension. Am J Respir Cell Mol Biol. 2020 Dec;63(6):843-855. doi: 10.1165/rcmb.2019-0317OC.
26. Kashimura O, Sakai A. Effects of physical training on pulmonary arterial pressure during exercise under hypobaric hypoxia in rats Int J Biometeorol. 1991 Dec;35(4):214-21.
27. Keserü B, Barbosa-Sicard E, Schermuly RT, Tanaka H, Hammock BD, Weissmann N, Fisslthaler B, Fleming I. Hypoxia-induced pulmonary hypertension: comparison of soluble epoxide hydrolase deletion vs. Inhibition. Cardiovasc Res. 2010 Jan 1;85(1):232-40.
28. Kikuchi N, Satoh K, Kurosawa R, Yaoita N, Elias-Al-Mamun M, Siddique MAH, Omura J, Satoh T, Nogi M, Sunamura S, Miyata S, Saito Y, Hoshikawa Y, Okada Y, Shimokawa H. Selenoprotein P Promotes the Development of Pulmonary Arterial Hypertension: Possible Novel Therapeutic Target. Circulation. 2018 Aug 7;138(6):600-623.
29. Koyama M, Furuhashi M, Ishimura S, Mita T, Fuseya T, Okazaki Y, Yoshida H, Tsuchihashi K, Miura T. Reduction of endoplasmic reticulum stress by 4-phenylbutyric acid prevents the development of hypoxia-induced pulmonary arterial hypertension. Am J Physiol Heart Circ Physiol. 2014 May;306(9):H1314-23.
30. Lahm T, Frump AL, Albrecht ME, Fisher AJ, Cook TG, Jones TJ, Yakubov B, Whitson J, Fuchs RK, Liu A, Chesler NC, Brown MB. 17β-Estradiol mediates superior adaptation of right ventricular function to acute strenuous exercise in female rats with severe pulmonary hypertension. Am J Physiol Lung Cell Mol Physiol. 2016 Aug 1;311(2):L375-88.
31. Marsboom G, Toth PT, Ryan JJ, Hong Z, Wu X, Fang YH, Thenappan T, Piao L, Zhang HJ, Pogoriler J, Chen Y, Morrow E, Weir EK, Rehman J, Archer SL. Dynamin-related protein 1-mediated mitochondrial mitotic fission permits hyperproliferation of vascular smooth muscle cells and offers a novel therapeutic target in pulmonary hypertension. Circ Res. 2012 May 25;110(11):1484-97.
32. McCullough DJ, Kue N, Mancini T, Vang A, Clements RT, Choudhary G. Endurance exercise training in pulmonary hypertension increases skeletal muscle electron transport chain supercomplex assembly. Pulm Circ. 2020 May 18;10(2):2045894020925762.
33. Megalou AJ, Glava C, Oikonomidis DL, Vilaeti A, Agelaki MG, Baltogiannis GG, Papalois A, Vlahos AP, Kolettis TM. Transforming growth factor-beta inhibition attenuates pulmonary arterial hypertension in rats. Int J Clin Exp Med. 2010 Oct 23;3(4):332-40.
34. Megalou AJ, Glava C, Vilaeti AD, Oikonomidis DL, Baltogiannis GG, Papalois A, Vlahos AP, Kolettis TM. Transforming growth factor-β inhibition and endothelin receptor blockade in rats with monocrotaline-induced pulmonary hypertension. Pulm Circ. 2012 Oct;2(4):461-9.
35. Moreira-Gonçalves D, Ferreira R, Fonseca H, Padrão AI, Moreno N, Silva AF, Vasques-Nóvoa F, Gonçalves N, Vieira S, Santos M, Amado F, Duarte JA, Leite-Moreira AF, Henriques-Coelho T. Cardioprotective effects of early and late aerobic exercise training in experimental pulmonary arterial hypertension. Basic Res Cardiol. 2015 Nov;110(6):57.
36. Natali AJ, Fowler ED, Calaghan SC, White E. Voluntary exercise delays heart failure onset in rats with pulmonary artery hypertension. Am J Physiol Heart Circ Physiol. 2015 Aug 1;309(3):H421-4.
37. Neto-Neves EM, Brown MB, Zaretskaia MV, Rezania S, Goodwill AG, McCarthy BP, Persohn SA, Territo PR, Kline JA. Chronic Embolic Pulmonary Hypertension Caused by Pulmonary Embolism and Vascular Endothelial Growth Factor Inhibition. Am J Pathol. 2017 Apr;187(4):700-712.
38. Nogueira-Ferreira R, Moreira-Gonçalves D, Silva AF, Duarte JA, Leite-Moreira A, Ferreira R, Henriques-Coelho T. Exercise preconditioning prevents MCT-induced right ventricle remodeling through the regulation of TNF superfamily cytokines. Int J Cardiol. 2016 Jan 15;203:858-66.
39. Okumura K, Kato H, Honjo O, Breitling S, Kuebler WM, Sun M, Friedberg MK. Carvedilol improves biventricular fibrosis and function in experimental pulmonary hypertension. J Mol Med (Berl). 2015 Jun;93(6):663-74.
40. Pacagnelli FL, de Almeida Sabela AK, Okoshi K, Mariano TB, Campos DH, Carvalho RF, Cicogna AC, Vanderlei LC. Preventive aerobic training exerts a cardioprotective effect on rats treated with monocrotaline. Int J Exp Pathol. 2016 Jun;97(3):238-47.
41. Piao L, Fang YH, Parikh K, Ryan JJ, Toth PT, Archer SL. Cardiac glutaminolysis: a maladaptive cancer metabolism pathway in the right ventricle in pulmonary hypertension. J Mol Med (Berl). 2013 Oct;91(10):1185-97.
42. Piao L, Fang YH, Parikh KS, Ryan JJ, D'Souza KM, Theccanat T, Toth PT, Pogoriler J, Paul J, Blaxall BC, Akhter SA, Archer SL. GRK2-Mediated Inhibition of Adrenergic and Dopaminergic Signaling in Right Ventricular Hypertrophy Circulation. 2012 Dec 11;126(24):2859-69.
43. Piao L, Sidhu VK, Fang YH, Ryan JJ, Parikh KS, Hong Z, Toth PT, Morrow E, Kutty S, Lopaschuk GD, Archer SL. FOXO1-mediated upregulation of pyruvate dehydrogenase kinase-4 (PDK4) decreases glucose oxidation and impairs right ventricular function in pulmonary hypertension: therapeutic benefits of dichloroacetate. J Mol Med (Berl). 2013 Mar;91(3):333-46.
44. Prins KW, Tian L, Wu D, Thenappan T, Metzger JM, Archer SL. Colchicine Depolymerizes Microtubules, Increases Junctophilin-2, and Improves Right Ventricular Function in Experimental Pulmonary Arterial Hypertension. J Am Heart Assoc. 2017 May 31;6(6):e006195.
45. Ryan JJ, Marsboom G, Fang YH, Toth PT, Morrow E, Luo N, Piao L, Hong Z, Ericson K, Zhang HJ, Han M, Haney CR, Chen CT, Sharp WW, Archer SL. PGC1α-mediated mitofusin-2 deficiency in female rats and humans with pulmonary arterial hypertension. Am J Respir Crit Care Med. 2013 Apr 15;187(8):865-78.
46. Schmidt C, Bovolini JA, Gonçalves N, Vasques-Nóvoa F, Andrade MDA, Santos M, Leite-Moreira A, Henriques-Coelho T, Duarte JA, Moreira-Gonçalves D. Exercise preconditioning prevents left ventricular dysfunction and remodeling in monocrotaline-induced pulmonary hypertension. Porto Biomed J. 2020 Sep 16;5(5):e081.
47. Schroll S, Arzt M, Sebah D, Nüchterlein M, Blumberg F, Pfeifer M. Improvement of bleomycin-induced pulmonary hypertension and pulmonary fibrosis by the endothelin receptor antagonist Bosentan. Respir Physiol Neurobiol. 2010 Jan 31;170(1):32-6.
48. Schroll S, Lange TJ, Arzt M, Sebah D, Nowrotek A, Lehmann H, Wensel R, Pfeifer M, Blumberg FC. Effects of simvastatin on pulmonary fibrosis, pulmonary hypertension and exercise capacity in bleomycin-treated rats. Acta Physiol (Oxf). 2013 Jun;208(2):191-201.
49. Schroll S, Sebah D, Wagner M, Popara V, Pfeifer M, Blumberg F. Improvement of exercise capacity in monocrotaline-induced pulmonary hypertension by the phosphodiesterase-5 inhibitor Vardenafil. Respir Physiol Neurobiol. 2013 Mar 1;186(1):61-4.
50. Sengul A, Arkan S, Vural C, Özer C, Bayrak B, Tas A, Kocak N, Aka U. Physiological and pathological effects of diminazene on pulmonary hypertension: A controlled rat model study. Int J Clin Exp Med. 2016; 9: 909-917.
51. Silva AF, Faria-Costa G, Sousa-Nunes F, Santos MF, Ferreira-Pinto MJ, Duarte D, Rodrigues I, Tiago Guimarães J, Leite-Moreira A, Moreira-Gonçalves D, Henriques-Coelho T, Negrão R. Anti-Remodeling Effects of Xanthohumol-Fortified Beer in Pulmonary Arterial Hypertension Mediated by ERK and AKT Inhibition. Nutrients. 2019 Mar 9;11(3):583.
52. Silva FJ, Drummond FR, Fidelis MR, Freitas MO, Leal TF, de Rezende LMT, de Moura AG, Carlo Reis EC, Natali AJ. Continuous Aerobic Exercise Prevents Detrimental Remodeling and Right Heart Myocyte Contraction and Calcium Cycling Dysfunction in Pulmonary Artery Hypertension. J Cardiovasc Pharmacol. 2021 Jan 1;77(1):69-78. doi: 10.1097/FJC.0000000000000928.
53. Soares LL, Drummond FR, Rezende LMT, Lopes Dantas Costa AJ, Leal TF, Fidelis MR, Neves MM, Prímola-Gomes TN, Carneiro-Junior MA, Carlo Reis EC, Natali AJ. Voluntary running counteracts right ventricular adverse remodeling and myocyte contraction impairment in pulmonary arterial hypertension model. Life Sci. 2019 Dec 1;238:116974.
54. Souza-Rabbo MP, Silva LF, Auzani JA, Picoral M, Khaper N, Belló-Klein A. Effects of a chronic exercise training protocol on oxidative stress and right ventricular hypertrophy in monocrotaline-treated rats. Clin Exp Pharmacol Physiol. 2008 Aug;35(8):944-8. doi: 10.1111/j.1440-1681.2008.04936.x. Epub 2008 Apr 21. PMID: 18430062.
55. Suen CM, Chaudhary KR, Deng Y, Jiang B, Stewart DJ. Fischer rats exhibit maladaptive structural and molecular right ventricular remodelling in severe pulmonary hypertension: a genetically prone model for right heart failure. Cardiovasc Res. 2019 Mar 15;115(4):788-799.
56. Vieira JS, Cunha TF, Paixão NA, Dourado PM, Carrascoza LS, Bacurau AVN, Brum PC. Exercise intolerance establishment in pulmonary hypertension: Preventive effect of aerobic exercise training. Life Sci. 2020 Nov 15;261:118298.
57. Weissmann N, Peters DM, Klöpping C, Krüger K, Pilat C, Katta S, Seimetz M, Ghofrani HA, Schermuly RT, Witzenrath M, Seeger W, Grimminger F, Mooren FC. Structural and functional prevention of hypoxia-induced pulmonary hypertension by individualized exercise training in Mouse. Am J Physiol Lung Cell Mol Physiol. 2014 Jun 1;306(11):L986-95.
58. Willis GR, Fernandez-Gonzalez A, Reis M, Yeung V, Liu X, Ericsson M, Andrews NA, Mitsialis SA, Kourembanas S. Mesenchymal stromal cell-derived small extracellular vesicles restore lung architecture and improve exercise capacity in a model of neonatal hyperoxia-induced lung injury. J Extracell Vesicles. 2020 Jul 13;9(1):1790874. doi: 10.1080/20013078.2020.1790874.
59. Wong MJ, Kantores C, Ivanovska J, Jain A, Jankov RP. Simvastatin prevents and reverses chronic pulmonary hypertension in newborn rats via pleiotropic inhibition of RhoA signaling. Am J Physiol Lung Cell Mol Physiol. 2016 Nov 1;311(5):L985-L999.
60. Wu J, Pan W, Wang C, Dong H, Xing L, Hou J, Fang S, Li H, Yang F, Yu B. H(2)S attenuates endoplasmic reticulum stress in hypoxia-induced pulmonary artery hypertension. Biosci Rep. 2019 Jul 8;39(7):BSR20190304.
61. Wunderlich C, Schmeisser A, Heerwagen C, Ebner B, Schober K, Braun-Dullaeus RC, Schwencke C, Kasper M, Morawietz H, Strasser RH. Chronic NOS inhibition prevents adverse lung remodeling and pulmonary arterial hypertension in caveolin-1 knockout Mouse. Pulm Pharmacol Ther. 2008;21(3):507-15.
62. Zimmer A, Teixeira RB, Bonetto JH, Siqueira R, Carraro CC, Donatti LM, Hickmann A, Litvin IE, Godoy AE, Araujo AS, Colombo R, Belló-Klein A. Effects of aerobic exercise training on metabolism of nitric oxide and endothelin-1 in lung parenchyma of rats with pulmonary arterial hypertension. Mol Cell Biochem. 2017 May;429(1-2):73-89.
